# Novel and Conserved Protein Macoilin Is Required for Diverse Neuronal Functions in *Caenorhabditis elegans*

**DOI:** 10.1371/journal.pgen.1001384

**Published:** 2011-05-12

**Authors:** Akiko Miyara, Akane Ohta, Yoshifumi Okochi, Yuki Tsukada, Atsushi Kuhara, Ikue Mori

**Affiliations:** 1 Laboratory of Molecular Neurobiology, Department of Molecular Biology, Graduate School of Science, Nagoya University, Nagoya, Japan; 2 CREST, Japan Science and Technology Agency, Saitama, Japan; University of California San Diego, United States of America

## Abstract

Neural signals are processed in nervous systems of animals responding to variable environmental stimuli. This study shows that a novel and highly conserved protein, macoilin (MACO-1), plays an essential role in diverse neural functions in *Caenorhabditis elegans*. *maco-1* mutants showed abnormal behaviors, including defective locomotion, thermotaxis, and chemotaxis. Expression of human macoilin in the *C. elegans* nervous system weakly rescued the abnormal thermotactic phenotype of the *maco-1* mutants, suggesting that macoilin is functionally conserved across species. Abnormal thermotaxis may have been caused by impaired locomotion of *maco-1* mutants. However, calcium imaging of AFD thermosensory neurons and AIY postsynaptic interneurons of *maco-1* mutants suggest that macoilin is required for appropriate responses of AFD and AIY neurons to thermal stimuli. Studies on localization of MACO-1 showed that *C. elegans* and human macoilins are localized mainly to the rough endoplasmic reticulum. Our results suggest that macoilin is required for various neural events, such as the regulation of neuronal activity.

## Author Summary

Any animals, including humans, have to be capable of properly sensing and responding to various environmental stimuli for survival and reproduction. Environmental stimuli are evaluated and, based on past experiences, converted to produce appropriate adaptive behaviors. Even the small, free-living soil nematode *Caenorhabditis elegans* can sense diverse environment stimuli using a nervous system that consists of only 302 neurons. *C. elegans* exhibit thermotaxis that allows them to remember ambient temperatures and use this information as a cue to seek and remain near food sources. We show here that a novel and conserved protein, MACO-1, is important for proper execution of thermotaxis by *C. elegans*. The *maco-1* gene was originally identified in a novel thermotaxis-defective mutant. Abnormal thermotaxis of *maco-1* mutants was weakly but apparently rescued by expressing human MACO-1, suggesting that MACO-1 is functionally conserved among species. MACO-1 protein is required for appropriate regulation of neuronal activity, and the activity of neurons required for thermotaxis is impaired in *maco-1* mutants. This analysis provides an important basis for the function of the macoilin family.

## Introduction

Genome projects have determined DNA sequences of various organisms and identified locations of predicted genes in genomes. Surprisingly, numbers of genes are relatively similar across evolutionally divergent animal species. For example, estimated gene numbers are 20000–25000 in human [1], 14000 in *Drosophila melanogaster* [2], and 19000 in *Caenorhabditis elegans* [3]. Of the estimated genes in *C. elegans*, more than 40 percent of the predicted protein products show significant matches to those from other animals [3]. Despite this species homology, functions of these genes are poorly understood. Therefore, further characterization of these proteins could lead to novel insights about important cellular processes, such as gene regulation, protein maturation, signal transduction, and intracellular transport.

**Figure 1:**
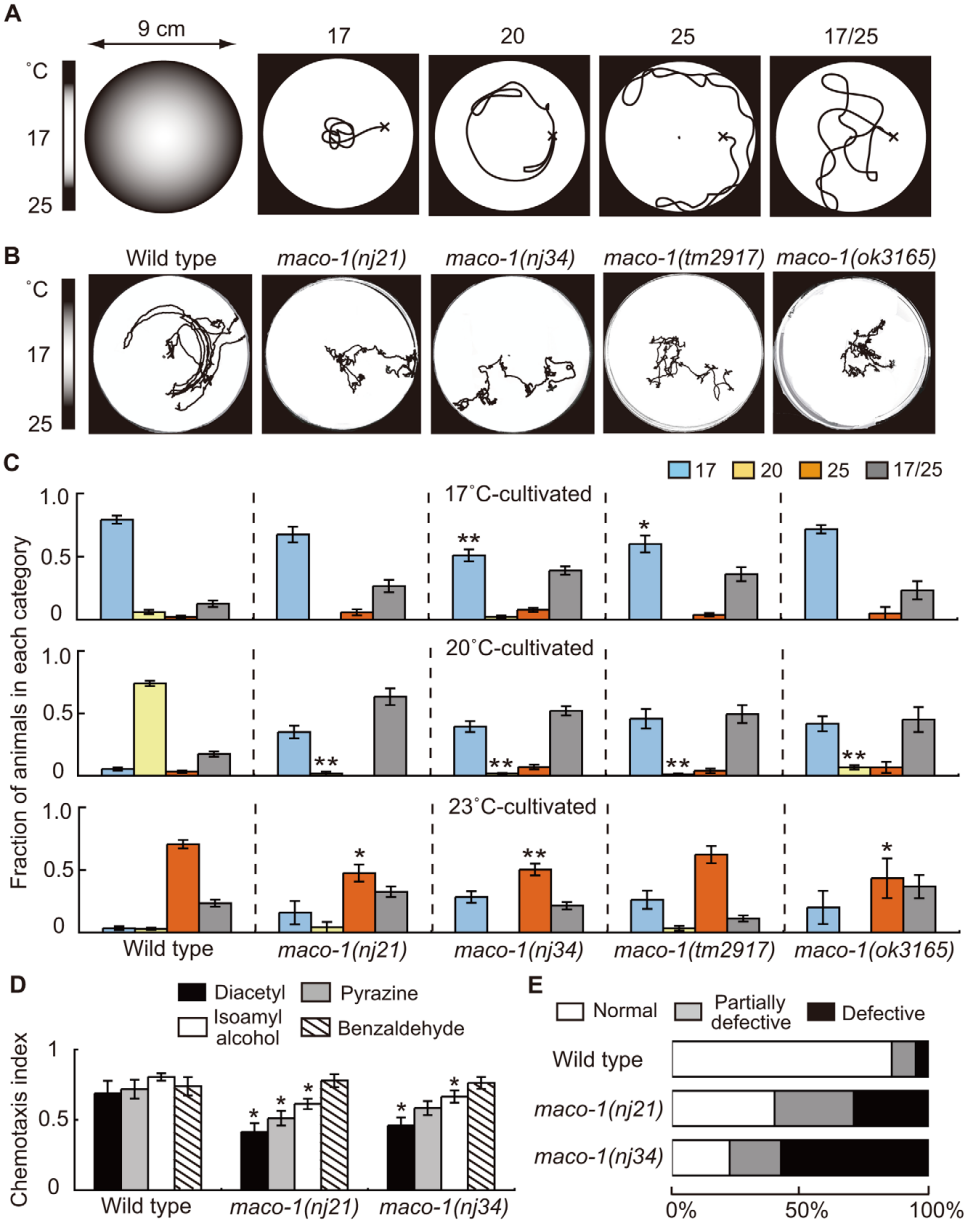
Phenotype of *maco-1*. (A) Thermotaxis assay with a radial temperature gradient. (Left panel of A) The center of the assay plate (a 9 cm-diameter petri dish) is approximately 17°C and the periphery is approximately 25°C. Tracks of animals were categorized into four groups after one-hour of free movement. Animals that moved to the 17°C region (center) were classified as ‘17’. Animals that moved to the 20°C region or 25°C region (periphery) were classified as ‘20’ or ‘25’, respectively. Animals that moved randomly around both 17°C and 25°C regions were classified as ‘17/25’. (B) Tracks of wild-type and *maco-1* mutant animals showing thermotaxis behaviors on a radial temperature gradient ranging from 17°C (center) to 25°C (periphery). Animals were grown at 20°C. (C) Thermotaxis of wild-type and *maco-1* mutant animals cultivated at 17°C (upper graph), 20°C (middle graph) and 23°C (lower graph). n = 60–380. Single and double asterisks indicate p<0.05 and p<0.01 by ANOVA with a Dunnett's post hoc test, respectively. (D) Chemotaxis of wild-type and *maco-1* mutant animals for odorants. Odorant concentrations are described in the Materials and Methods section. For each assay, 50–300 animals were analyzed. A single asterisk indicates p<0.05 compared to wild-type by ANOVA with a Dunnett's post hoc test. Bars represent the mean of three independent assays with error bars showing the standard error of the mean (SEM). (E) Chemotaxis to sodium chloride (NaCl). For each genotype, 40–120 animals were individually assayed. The phenotypic categories are described in the Materials and Methods section. Mean fractions categorized as ‘Normal’ were 0.86±0.05 among wild-type animals, 0.40±0.04 in *maco-1(nj21)* mutants and 0.23±0.18 in *maco-1(nj34)* mutants. Statistically significant differences were observed between the fraction of ‘Normal’ of wild-type and *maco-1* mutants (p<0.01 by ANOVA with a Dunnett's post hoc test).

Animals use the nervous system to detect stimuli in the surrounding environment. These stimuli are then assessed based on past experiences, and converted into adaptive behaviors. Many neurons are required to act in coordination to execute these processes. However, it is difficult to address individual neuronal function in relation to other neurons in neural circuits because animal brains generally consist of enormous numbers of neurons. The anatomically characterized nervous system of *C. elegans* is composed of only 302 neurons [4], making it an effective model for studying neuronal function in circuits. Despite their simple nervous systems, *C. elegans* can sense an array of environmental stimuli and produce appropriate behavioral outputs. For example, worms remember cultivation temperatures in association with food conditions [5]–[7]. This thermotaxis behavior can be divided into two behavioral aspects. First, after cultivation *C. elegans* with food at a certain temperature for several hours, they will migrate to this cultivation temperature along a temperature gradient [5]–[7]. Previous studies have suggested that this migratory behavior could be explained by the following two opposing strategies: migrations up thermal gradients when located at temperatures below the cultivation temperature (i.e., thermophilic drive); and migrations down thermal gradients when at a higher temperature (i.e., cryophilic drive) [5]–[7]. Work by several groups has been unable to confirm thermophilic drive in *C. elegans* [8]–[11]. However, recent theoretical and experimental studies have shown that the steepness of the thermal gradient drastically changes migration behavior [12], [13]. The second behavioral aspect of thermotaxis involves worms leaving isothermal tracks (IT) on a temperature gradient as they migrate to the cultivation temperature associated with food (Figure 1B and [5], [6]). Previous reports have suggested that the neuron-specific calcium sensor-1 (NCS-1) plays an important role in optimizing IT [14]. Specifically, reports showed that a knockout phenotype of *ncs-1* animals could be rescued by introducing a wild-type NCS-1 into the AIY interneuron, which is a component of the thermotaxis neural circuit, and that NCS-1 overexpression enhanced the frequency of IT behavior [14].

The thermotaxis phenotype of *C. elegans*, which was achieved by laser-ablating specific neurons, has been examined previously, and results led to the hypothesis of a neural circuit for thermotaxis [6]. This neural circuit is presently one of the simplest circuits to regulate animal behavior. By capitalizing on this simple structure, we expect that the study of thermotaxis behavior will reveal molecular mechanisms of thermosensation and concepts of neural plasticity and neuronal function. Several molecular components related to thermotaxis behavior have been identified. The three guanylyl cyclases, GCY-8, GCY-18 and GCY-23, which are expressed in AFD thermosensory neurons, appear to redundantly produce cyclic guanosine monophosphate (cGMP) as a second messenger. The cGMP-dependent cation channel, which is composed of TAX-2 and TAX-4, is crucial in activating AFD neurons [15]–[17]. TAX-6 (calcineurin) and TTX-4 (PKC-1; nPKC-epsilon/eta), both of which act as negative regulators of AFD thermosensory signaling, have also been reported [18], [19]. Calcium imaging using the genetically encoded calcium indicator cameleon can display fluorescence changes from variable intracellular calcium concentrations [20]. This imaging has shown that AFD neurons respond to thermal stimuli in a cultivation temperature-dependent manner [21]. This response occurred even after disconnecting the AFD dendrites from their cell bodies [22], indicating that temperature memories are stored in AFD neurons. Consistent with findings in AFD neurons, recent calcium imaging analyses also suggest that AWC thermosensory neurons can also store temperature memories [23], [24].

In this study, we found that the novel protein MACO-1 is required for diverse neuronal functions in *C. elegans*. *maco-1/ttx-8* mutants exhibited abnormalities in thermotaxis and chemotaxis to odorants and sodium chloride (NaCl). The *maco-1* gene encodes a conserved novel protein that is expressed in many neurons. A previous study reported that the mouse ortholog of MACO-1, macoilin (C61), showed neuron-specific expression [25], although its function remained largely unknown. Rescue experiments revealed that simultaneous expression of MACO-1 in AFD, AIY and AIZ neurons of *maco-1* mutants is required for thermotaxis behavior. We also found that *maco-1* mutants showed abnormal locomotion resulting from frequent turns. Although defective locomotion of *maco-1* mutants might have caused abnormal thermotaxis, calcium imaging indicated that MACO-1 is required for activation of AFD and AIY neurons. *In vivo* co-localization studies have shown that both MACO-1 and human macoilin are localized to the rough endoplasmic reticulum (rER). All together, our results suggest that macoilin is required for a variety of intracellular events that influence basic neuronal function across species.

## Results

### *maco-1/ttx-8* mutants showed prominent thermotaxis defects and slight chemotaxis defects

To elucidate molecular mechanisms of thermotaxis, we performed a genetic screen and isolated several mutants that were thermotaxis defective [19]. Two of these mutants, *maco-1(nj21)* and *maco-1(nj34)*, were isolated as novel mutants. Two other mutant alleles, *maco-1(tm2917)* and *maco-1(ok3165)*, were isolated by the National Bioresource Project (Japan) and the *C. elegans* Gene Knockout Consortium, respectively. All four alleles were genetically recessive (data not shown). Despite obvious defects in thermotaxis after cultivation at 20°C (Figure 1B and 1C), *maco-1(nj21)* and *maco-1(nj34)* mutants were slightly defective in chemotaxis to odorants (Figure 1D and Figure S1) or to NaCl (Figure 1E). These results suggest that MACO-1 is necessary for animals to respond normally to various stimuli, including temperature, odorants and NaCl. Strong thermotaxis defects in *maco-1* mutants after cultivation at 20°C suggest that MACO-1 may play an important role in thermotaxis, especially after cultivation at 20°C. Alternatively, the thermotaxis defects in *maco-1* mutants could have been more easily detected in thermotaxis assays for 20°C-grown animals (20°C TTX assay) than 17°C or 23°C TTX assays. Specifically, during 20°C TTX assays, animals must migrate and remain in the 20°C region, which is shown as the light grey zone in the left schematic of Figure 1A. By contrast, during 17°C and 23°C TTX assays, animals can achieve thermotactic behaviors by migrating to 17°C and 23°C regions at the center and the periphery of a 9 cm plate, respectively. These areas are places where animals tend to remain due to the nature of assay plates and non-linear temperature gradients.

### The *maco-1* gene encodes a novel protein

**Figure 2:**
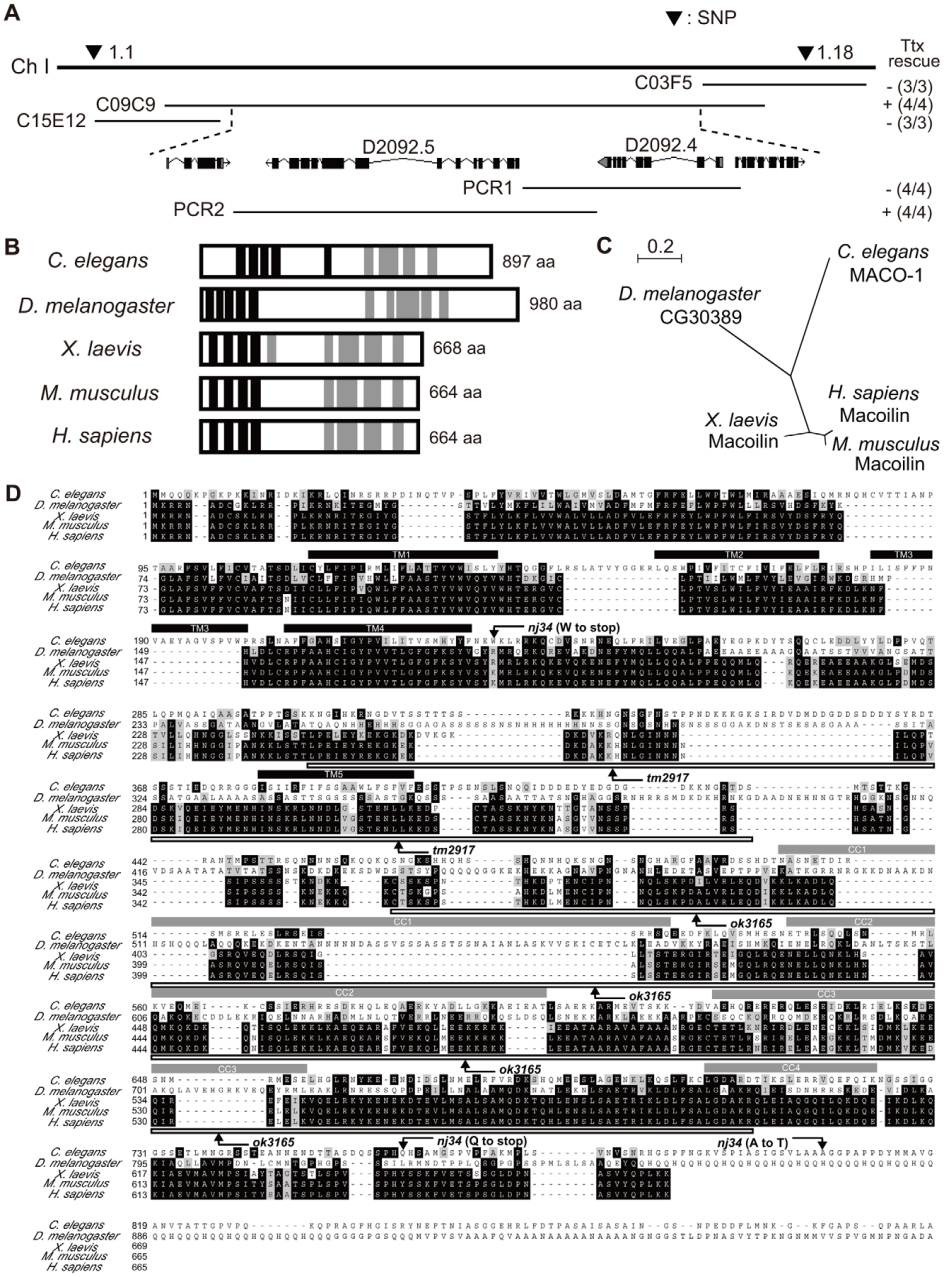
Genetic and molecular analysis of *maco-1*. (A) Position of *maco-1* on chromosome I. Arrowheads show locations of single nucleotide polymorphisms (SNPs). Results of rescue experiments among thermotaxis-defective *maco-1* mutants are indicated as + (rescued) or − (not rescued) on the right side. Numbers in parentheses indicate the fraction of rescued lines. For each genotype, 120–180 animals were individually assayed for thermotaxis. The *maco-1* gene was mapped to a 100–kb region between two SNPs (1.1 and 1.18) on chromosome I. We conducted rescue experiments using overlapping cosmids to encompass this 100-kb region. (B) Predicted structure of *maco-1* gene family. Black and gray boxes indicate transmembrane domains and coiled coil domains, respectively. Numbers of amino acids are shown on the right. (C) Unrooted dendrogram of MACO-1. The dendrogram was generated using the freely available program, Clustal W [52] (http://clustalw.genome.jp/) and NJplot [53]. (D) Comparison of sequences of MACO-1 and orthologs of MACO-1 in other species and mutation sites of *maco-1*. Black and gray boxes highlight identical and similar residues, respectively. Black bars and gray bars above the sequences indicate predicted transmembrane domains (TM1–TM5) and coiled coil domains (CC1–CC4) of MACO-1 in *C. elegans*, respectively. Boxes below the amino-acid alignment correspond to mutation sites of *tm2917* and *ok3165*.

The *maco-1* gene was identified by mapping it with the snip-single nucleotide polymorphisms (SNPs) method [26] and conducting subsequent rescue experiments with genomic or polymerase chain reaction (PCR) fragments. Defective thermotaxis of *maco-1* mutants was rescued by introduction of the cosmid C09C9. However, thermotaxis was not rescued by the cosmid C15E12 or C03F5 (Figure 2A), suggesting that *maco-1* is located in the region between cosmids C15E12 and C03F5 within the cosmid C09C9. In addition, a PCR fragment containing D2092.4 gene (PCR1) did not rescue abnormal thermotaxis behavior of *maco-1* mutants. However, another PCR fragment containing D2092.5 gene (PCR2) could rescue this behavior (Figure 2A), suggesting that D2092.5 is identical to the *maco-1* gene. D2092.5/*maco-1* gene encodes a novel protein that was predicted to have five transmembrane domains in the N-terminus and four coiled coil domains in the C-terminus as determined by the TMHMM Server v. 2.0 [27] and by COILS server [28], respectively (Figure 2B). Blast searches of the U.S. National Center for Biotechnology Inventory (NCBI) inventory identified *maco-1* orthologs in several species, including the invertebrates *Drosophila melanogaster*, and vertebrates *Xenopus laevis*, *Mus musculus* and *Homo sapiens* (Figure 2B, 2C and Table S1). The *nj21* mutant has a G-to-A substitution at the splice acceptor site of the 9th intron. The *nj34* mutant has two nonsense mutations (W231stop and Q761stop) and a missense mutation (A805T) (Figure 2D). The *tm2917* mutant has a 780-base pair (bp) deletion and 1-bp insertion just before the first coiled coil domain (Figure 2D). The *ok3165* mutant has a 972-bp deletion and 1-bp insertion in the coiled coil region (Figure 2D).

### MACO-1 is mainly localized to the rER

**Figure 3:**
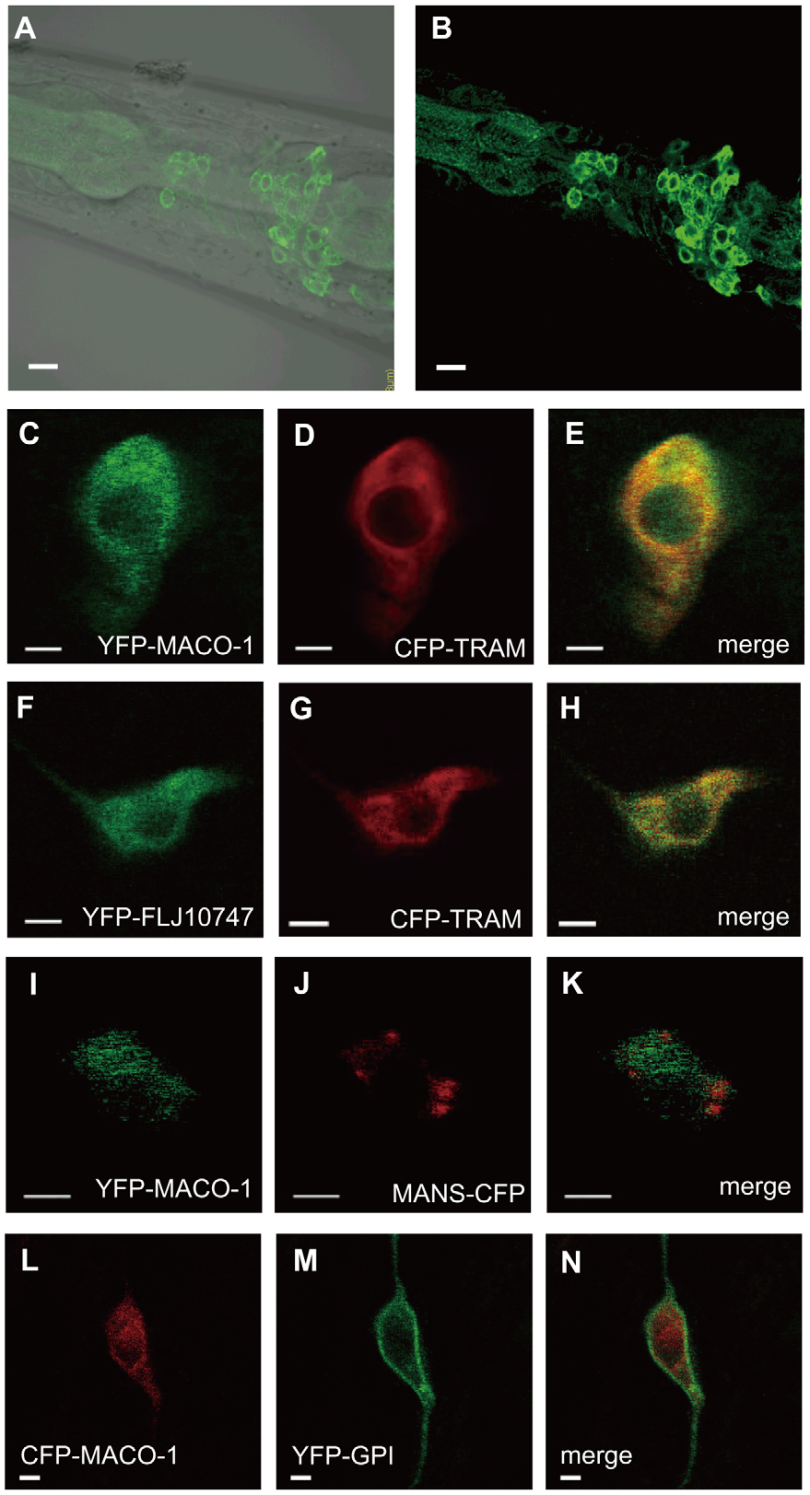
Subcellular localization of MACO-1. (A, B) Subcellular localization of the *almost full-length genomic maco-1::GFP*, including predicted transmembrane domains and coiled coil domains in wild-type animals. (A) Merged differential interference contrast (DIC) and green fluorescent protein (GFP) images. (B) GFP image. Anterior is to the left and dorsal is up. (C–N) Co-expression of MACO-1 or human ortholog of MACO-1 (FLJ10747) fused to yellow fluorescent protein (YFP) or cyan fluorescent protein (CFP) with organelle markers in AFD thermosensory neurons of wild-type animal. The following markers were used: CFP-tagged translocating chain-associating membrane protein (CFP::TRAM) as a marker of rough endoplasmic reticulum (rER); CFP-tagged mannosidase (MANS::CFP) as a marker of Golgi bodies; membrane anchored YFP (YFP::GPI) as a marker of plasma membranes [29]. (C–E) Co-expression of YFP::MACO-1 with CFP::TRAM. (F–H) Co-expression of YFP::FLJ10747 with CFP::TRAM. (I–K) Co-expression of YFP::MACO-1 with MANS::CFP. (L–N) Co-expression of YFP::GPI with CFP::MACO-1. Scale bars: A and B = 5 µm; C–N = 2 µm.

To study subcellular localization of MACO-1, we introduced the *almost full-length genomic maco-1::GFP*, which can rescue abnormal thermotaxis of *maco-1* mutants (Figure S2A), into wild-type animals and observed green fluorescent protein (GFP) fluorescence. Expression of this fusion protein was observed in the cell body but not in the neurite (Figure 3A and 3B). Because MACO-1 was found in the cell body and is predicted to be a membrane protein, we investigated whether the protein is localized to organelles, such as the ER or the Golgi bodies. We made several transgenic lines co-expressing cyan fluorescent protein (CFP)- or yellow fluorescent protein (YFP)-fused MACO-1 and one of the following organelle markers: Translocating-chain associating membrane protein (CFP::TRAM) as a rER marker; Mannosidase (MANS::CFP) as a Golgi marker and membrane anchored YFP (YFP::GPI) as a plasma membrane marker [29]. We found that functional YFP::MACO-1 fusion protein was co-localized with the rER marker, CFP::TRAM in AFD neurons (Figure 3C–3E, Figure S2C and Text S1). Similarly, YFP fusion protein with human macoilin, YFP::FLJ10747, was also co-localized with CFP::TRAM in AFD neurons (Figure 3F–3H). Further, little to no co-localization of YFP::MACO-1 was observed with the Golgi marker, MANS:CFP (Figure 3I–3K), and CFP::MACO-1 was not co-localized with the plasma membrane marker, YFP::GPI (Figure 3L–3N). These results suggest that MACO-1 is mainly localized to the rER. Consistent with these findings, Arellano-Carbajal et al. showed that an antibody against MACO-1 was predominantly localized to the rER of *C. elegans* neurons (Arellano-Carbajal et al., personal communication).

### MACO-1 functions in neurons and is conserved across species

**Figure 4:**
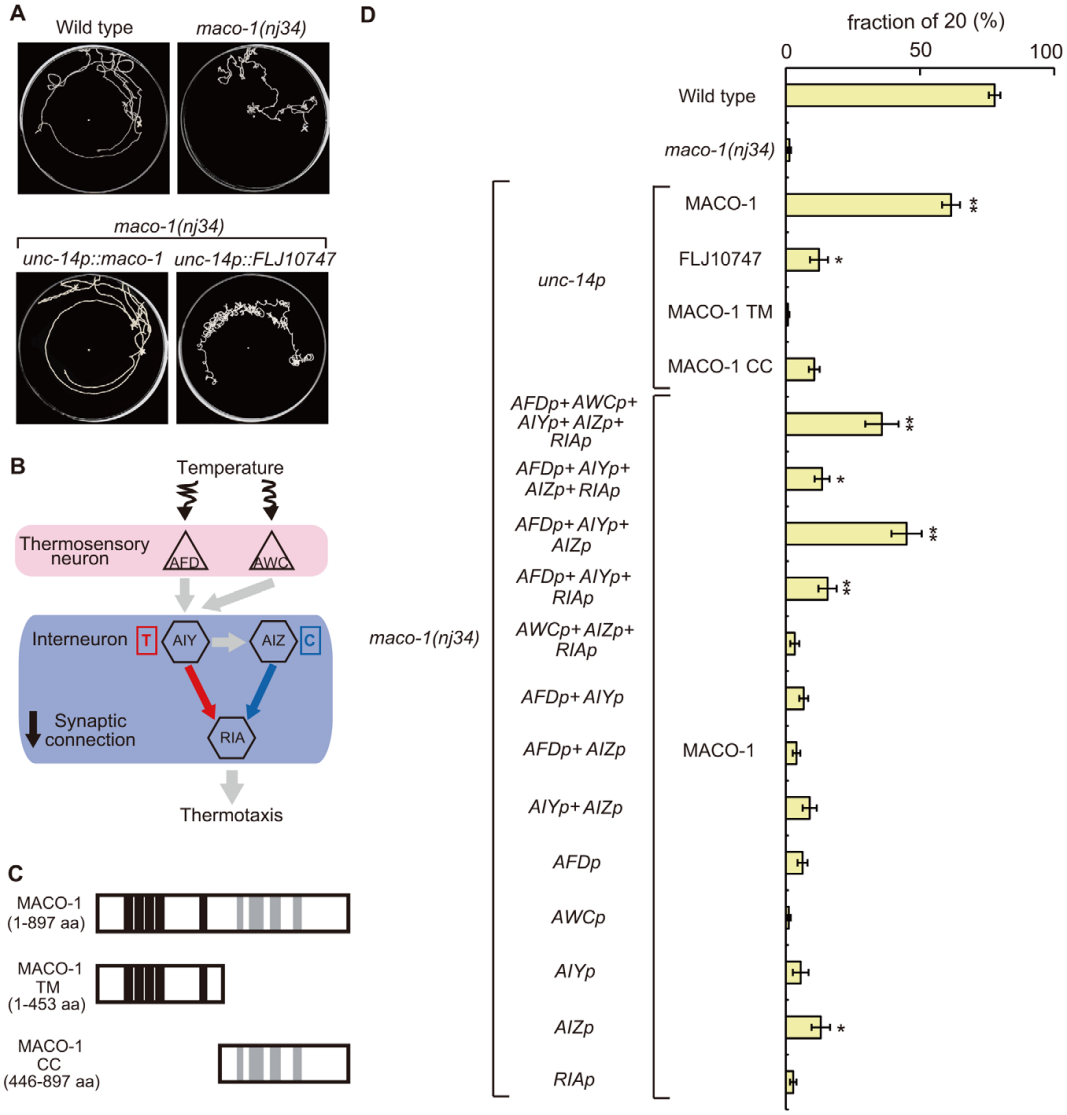
Cell-specific rescue experiments for thermotaxis defects in *maco-1(nj34)* mutants. (A) Tracks of animals showing thermotaxis behavior when grown at 20°C. Upper left panel is wild-type animal. Upper right panel is *maco-1(nj34)* mutant. Lower left panel is *maco-1(nj34)* expressing *maco-1* cDNA in almost all neurons. Lower right panel is *maco-1(nj34)* expressing human ortholog of MACO-1 in almost all neurons. The *unc-14* promoter was used for expression in almost all neurons. (B) The proposed neural circuit model for thermotaxis [6]. Temperature is sensed by AFD and AWC sensory neurons. The AIY-RIA circuit is involved in thermophilic behavior (i.e., movements to temperatures higher than the cultivation temperature indicated as ‘T’). The AIZ-RIA circuit is involved in cryophilic behavior (i.e., movements to temperatures lower than the cultivation temperature indicated as ‘C’). RIA neurons are proposed to integrate these signals. (C) Truncated forms of MACO-1. MACO-1 TM (from 1 to 453 amino acids) lacks coiled coil regions and MACO-1 CC (from 446 to 897 amino acids) lacks transmembrane regions. (D) Rescue of the thermotaxis defect in *maco-1* mutants by specific expression of *maco-1* cDNA, human ortholog of *maco-1* cDNA, MACO-1 TM or MACO-1 CC in almost all neurons using the *unc-14* promoter. Specific observed expressions of *maco-1* cDNA using several cell-specific promoters. Expression patterns of each promoter are listed in Table S2. Bars show the percentage of animals that moved to the cultivation temperature (20°C). Error bar indicates the standard error of the mean (SEM). Asterisks indicate statistically significant differences between fractions of 20 of 20°C-cultivated transgenic strains relative to fractions of 20 of 20°C-cultivated *maco-1* mutants. One asterisk denotes statistical significance at p<0.05 and two asterisks denote statistical significance at p<0.01 (ANOVA with a Dunnett's post hoc test; n = 120–680).

To investigate the expression pattern of *maco-1*, we constructed the *maco-1 promoter::GFP* reporter that used an approximately 5-kilobase (kb) upstream region from the ATG initiation codon as a *maco-1* promoter. This construct was then introduced into wild-type animals. We found that strong expression was observed in many neurons (Figure S3A and Text S1), while very weak expression was also detected in tissues. Thermotaxis defects were consistently rescued when *maco-1* cDNA was expressed in almost all neurons of *maco-1* mutants using the *unc-14* promoter, indicating that MACO-1 acts in neurons (lower left panel of Figure 4A and Figure 4D).

To examine whether MACO-1 is conserved functionally among species, we introduced FLJ10747, a predicted human ortholog of MACO-1, into almost all neurons of *maco-1* mutants using the *unc-14* promoter. We then evaluated the thermotaxis phenotype of the transgenic strain (lower right panel of Figure 4A, Figure 4D, Figure S2B and Text S1). FLJ10747 rescued, albeit weakly, abnormal thermotaxis phenotype of *maco-1* mutants, suggesting that MACO-1 is functionally conserved across species.

### Both transmembrane domains and coiled coil domains are required for appropriate function of MACO-1

MACO-1 was predicted to have transmembrane domains in the N-terminus and coiled coil domains in the C-terminus (Figure 2B). To examine whether these regions are necessary for MACO-1 function, we constructed two truncated proteins, MACO-1 TM (lacks coiled coil region) and MACO-1 CC (lacks transmembrane region), and conducted rescue experiments using each protein (Figure 4C and 4D). Expression of MACO-1 TM did not rescue in nearly all neurons of *maco-1* mutants using the *unc-14* promoter, while MACO-1 CC slightly rescued the abnormal thermotaxis of *maco-1* mutants (Figure 4D). We also conducted rescue experiments using YFP::MACO-1 TM or YFP::MACO-1 CC fusion proteins to address whether degradation of these truncated proteins or ER quality influenced rescue experiment results. We verified that YFP fluorescence was observed in both truncated fusion proteins (Figure S4J–S4M and Text S1). YFP::MACO-1 TM did not rescue and YFP::MACO-1 CC slightly rescued defective thermotaxis of *maco-1* mutants (Figure S2C and Text S1). We also found that YFP::MACO-1 TM appeared to localize to the rER (Figure S4D–S4F and Text S1). However, YFP::MACO-1 CC localization to the rER was minimal, but rather was localized to cell bodies and neurites (Figure S4G–S4I, S4L, S4M and Text S1). These results suggest that MACO-1 TM regions are necessary for appropriate MACO-1 localization, and MACO-1 CC regions are important for proper function of MACO-1.

### Proper function of MACO-1 is necessary for normal thermotaxis in many neurons

To identify cells that require MACO-1 activity for sensory behavior, *maco-1* cDNA was expressed by using various cell-specific promoters. Expression of MACO-1 in AFD, AWC, AIY, AIZ and RIA neurons, which are essential for thermotaxis behavior (Figure 4B and [6], [24]), partially rescued the thermotaxis defect of *maco-1* mutants (Figure 4D). By contrast, specific expression of *maco-1* cDNA in AFD, AWC, AIY AIZ or RIA neurons individually did not rescue or only slightly rescued the abnormal thermotaxis phenotype of *maco-1* mutants (Figure 4D). Thus, we propose that simultaneous expression of MACO-1 in multiple neurons of the thermotaxis neural circuit is necessary to rescue abnormal thermotaxis among *maco-1* mutants. We attempted but were unable to express MACO-1 in several combinations of neurons in *maco-1* mutants to find the minimum set of neurons required to ensure full rescue of thermotaxis behavior. However, the expression of MACO-1 in AFD, AIY and AIZ neurons rescued *maco-1* mutant defects similar to the expression of MACO-1 in AFD, AWC, AIY, AIZ and RIA neurons (Figure 4D). Notably, expression of MACO-1 in any other combinations of AFD, AWC, AIY, AIZ and RIA neurons only weakly rescued the defect (Figure 4D). All together, these results suggest that MACO-1 activity in AFD, AIY and AIZ neurons is required for thermotaxis behavior, and MACO-1 activity is needed in many neurons to achieve full rescue.

### Abnormal thermotaxis behavior is not attributable to indirect effects of defective locomotion in *maco-1* mutants

Cell-specific rescue experiments have implicated that a functional defect of MACO-1 in AFD, AIY and AIZ neurons caused defects in thermotaxis of *maco-1* mutants. However, we also observed that *maco-1* mutants changed their direction of movement more frequently than wild-type animals based on their tracks left during TTX assays (Figure 1B). This observation raised another possibility that locomotory defects secondarily causes the abnormal thermotaxis behavior of *maco-1* mutants.

**Figure 5:**
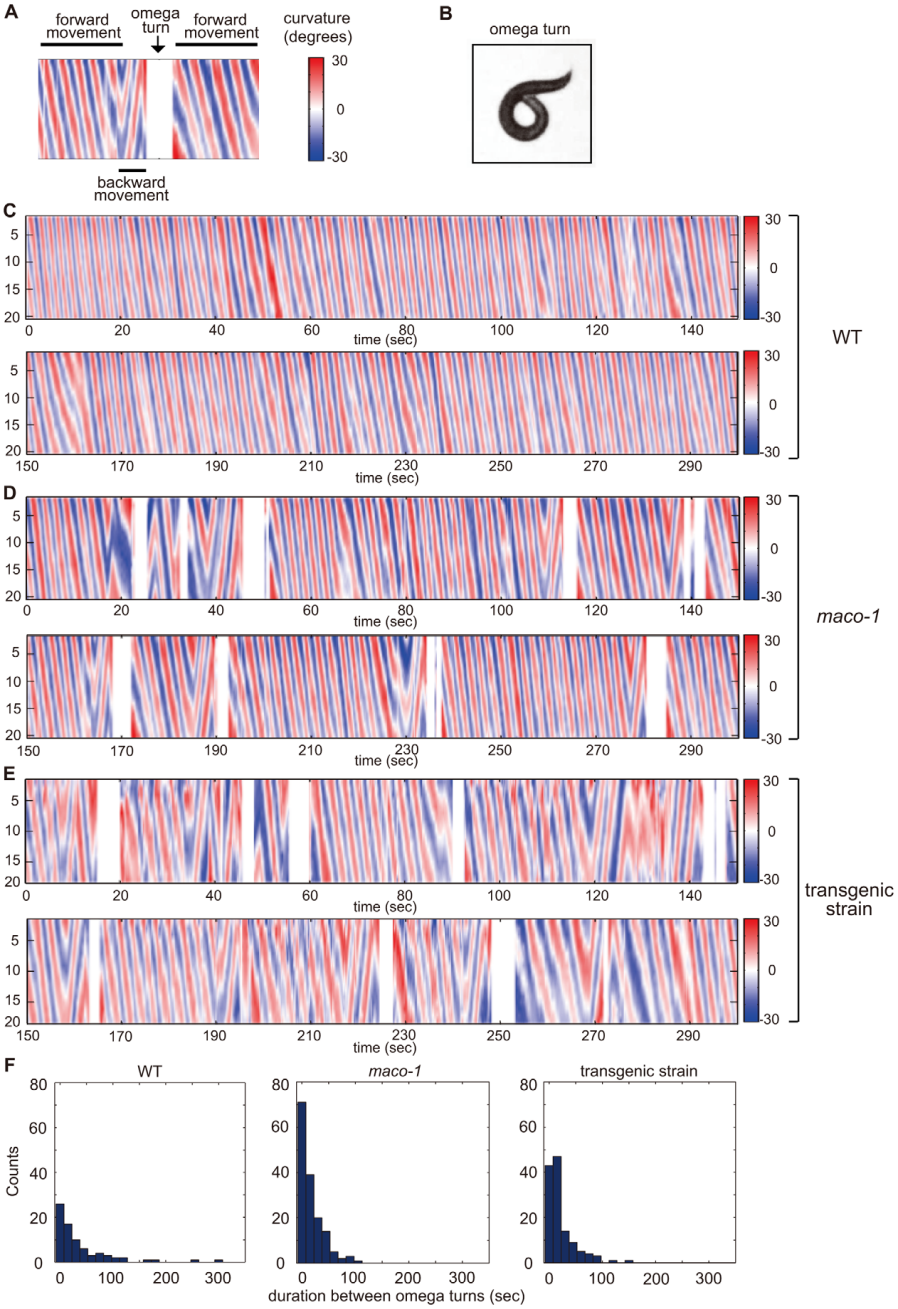
Curvature maps of animal body along the head-to-tail axis. (A) Examples of locomotor states. Black bars above and below the curvature map indicate the state of forward movement and backward movement, respectively. The state of omega turn was indicated as white space (arrow). Although animals sometimes showed modest changes in direction, it is not reflected in this curvature map. (B) An image of omega turn. The head of animal curls to touch its own body. (C–E) Curvature maps of individual animals. Well-fed single animals were placed on TTX plates. Observations began 10 min after transfer. Freely moving individual animals were captured by video camera for 30 min. The x-axis and y-axis indicate time and the position along animal midlines, respectively. The color-map to the right of the graph (numbered from −30 to 30) depicts curvatures (see the Materials and Methods section for calculation procedures). (C) Wild-type. (D) *maco-1(nj34)*. (E) *maco-1(nj34)* expressing *maco-1* cDNA in AFD, AWC, AIY, AIZ and RIA neurons. This transgenic strain showed a partially rescued phenotype in thermotaxis behavior (Figure 4D). (F) Duration between omega turns. Initial 1000-second video images of each animal were analyzed by image-processing software, MATLAB. n = 4 (wild-type), 3 (*maco-1*) and 2 (transgenic strain).

Spontaneous locomotion of *C. elegans* is roughly classified into two categories. The first category, runs, includes long series of sinusoidal-swimming movement. The second category, turns, is divided into two types. Omega turns involve the animal curling its head toward the tail and continues to propel forward. Reversals involve the animal moving backwards for several seconds and then moving forwards in a new direction [30], [31]. To quantify the potential locomotory defect, we constructed curvature maps of animal bodies along the head-to-tail axis using a tracking system and homemade MATLAB programs. Locomotion was represented by patterns in the curvature map (Figure 5A and 5C–5E). Wild-type animals spent most of their time moving forward (Figure 5C and left graph of 5F). Conversely, forward movement was interrupted by omega turns at short intervals among *maco-1* mutant animals (Figure 5D and middle graph of 5F). A similar tendency was observed in transgenic *maco-1* mutants (Figure 5E and left graph of 5F), whose abnormal thermotaxis was partially rescued by expression of *maco-1* cDNA in AFD, AWC, AIY, AIZ and RIA neurons (Figure 4D). This finding supports that defective locomotion does not cause abnormal thermotaxis in *maco-1* mutants.

### MACO-1 is required for proper activation of AFD and AIY neurons

**Figure 6:**
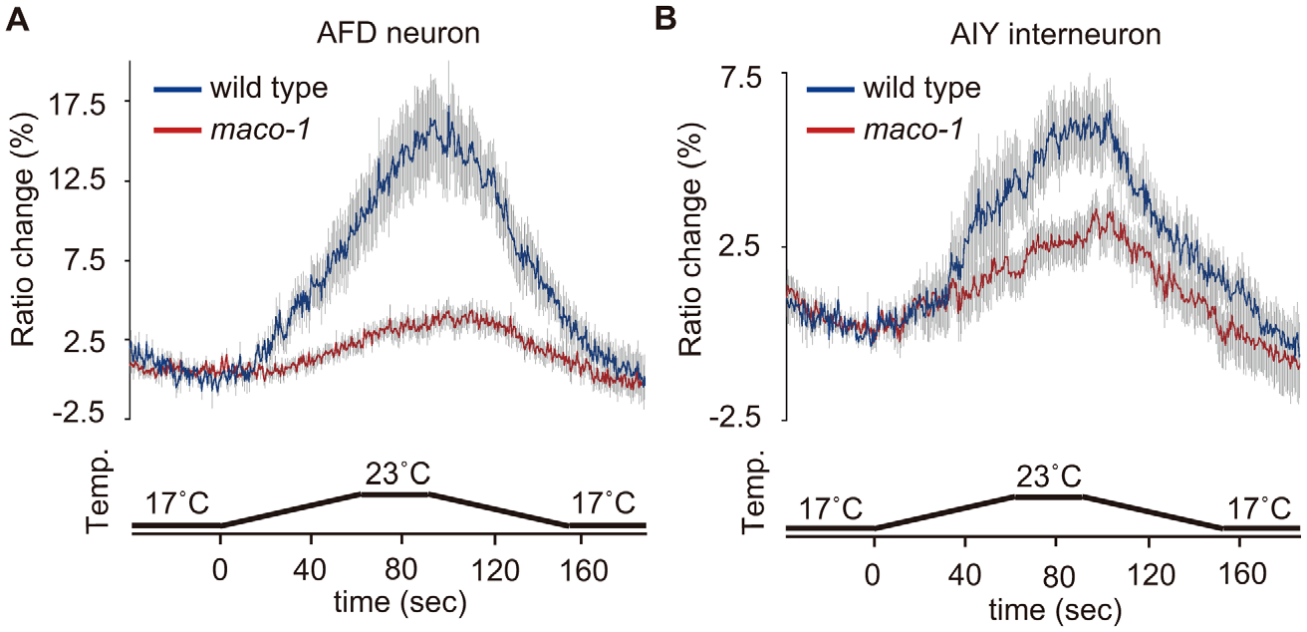
Calcium imaging of neurons in the thermotaxis circuit among wild-type or *maco-1* mutants. *In vivo* Ca^2+^ ratio imaging in individual genotypes cultivated at 20°C. (A) AFD thermosensory neuron. (B) AIY interneuron. Relative increases or decreases in the intracellular Ca^2+^ concentration were measured as increases or decreases in fluorescence of the yellow fluorescent protein/cyan fluorescent protein (YFP/CFP) of the cameleon protein ratio (ratio change). Blue and red traces represent the mean value of ratio changes of wild type and *maco-1(nj34)* animals, respectively, during temperature changes as indicated in the bottom panel of each graph (n = 10). Error bars (gray line) indicate the standard error of the mean (SEM). The average of the maximum ratio change from baseline ± SEM was as follows: 17.1±1.6 for wild-type (AFD); 4.3±0.8 for *maco-1* (AFD); 6.3±1.0 for wild-type (AIY) and 3.8±0.6 for *maco-1* (AIY).

Cell-specific rescue experiments indicated that MACO-1 activity, at least in AFD, AIY and AIZ neurons, is necessary for normal thermotaxis behavior (Figure 4D). To examine whether the MACO-1 functional defect influenced activities of these neurons, we conducted calcium imaging experiments and monitored responses of AFD and AIY neurons from *maco-1(nj34)* mutants to thermal stimuli. We found that the ratio change in AFD neurons of *maco-1(nj34)* mutants was significantly less than that of wild-type animals (Figure 6A). Previous work has shown that the ratio change in AFD neurons of *tax-4(p678)* mutants was hardly detected [21]. AFD neurons of the *tax-4(p678)* mutant do not respond properly to temperature because of defective cGMP-dependent cation channels composed of TAX-2 and TAX-4. The average of maximum ratio change from baseline ± SEM in *maco-1(nj34)* mutants was slightly larger (4.3±0.8) relative to *tax-4(p678)* mutants (3.3±0.6) [21] (Figure 6A), suggesting that AFD neuronal response to thermal stimuli among *maco-1(nj34)* mutants was not completely lost but partially reduced. Similarly, the ratio change in AIY neurons of *maco-1* mutants was also lower than that of wild-type animals (Figure 6B). Taken together, our data suggest that activities of both AFD and AIY neurons in *maco-1* mutants are partially decreased in response to thermal change, further indicating that MACO-1 is required for activation of AFD and AIY neurons. We also examined morphologies of these neurons in *maco-1* mutants by expressing GFP in AFD and AIY neurons to determine whether the decreased activity of AFD and AIY neurons was caused by developmental or functional defects. The AFD and AIY neurons in *maco-1* mutants appeared normal (Figure S3B, S3C and Text S1), suggesting that MACO-1 is not required for development but rather for proper function of AFD and AIY neurons.

## Discussion

### MACO-1 is required for proper activation of neurons

We found that proper function of MACO-1 in AFD, AIY and AIZ neurons is necessary for normal thermotaxis behavior. Consistent with this result, calcium imaging suggested that MACO-1 is required for accurate activation of AFD and AIY neurons. Rescue experiments also indicate that appropriate MACO-1 function in many other interneurons and motor neurons is probably required for full rescue of abnormal thermotaxis behavior. These results suggest that MACO-1 is required broadly for proper neural activation. Consistent with this postulate, *maco-1* mutants showed additional behavioral defects in chemotaxis and locomotion.

### MACO-1 likely plays a role in assisting fundamental neuronal function

Co-localization studies revealed that MACO-1 was mainly localized to the rER. The rER is generally thought to be required for protein synthesis, folding and translocation of secretory and transmembrane proteins. Thus, functional defects of MACO-1 in the rER might alter these events, resulting in abnormal phenotypes in *maco-1* mutants.

Previous studies have reported that Resistant to Inhibitors of Cholinesterase 3 (RIC-3) is localized to the ER and required for the maturation (the folding, assembly or transport) of nicotinic acetylcholine receptors [32]–[39]. RIC-3 is predicted to have two transmembrane domains followed by coiled-coil domains. Similar structural motifs and intracellular localization patterns of RIC-3 and MACO-1 suggest that MACO-1 might be involved in the maturation of membrane proteins like RIC-3. To explore this possibility, we observed the localization of several membrane proteins that could influence abnormal behaviors of *maco-1* mutants. We found slightly increased spacing between adjacent SNB-1::GFP puncta (presynaptic marker) in DD motor neurons of *maco-1* mutants compared with wild-type animals (Figure S5, Text S1 and [40], [41]). This change could be attributable to MACO-1 being directly associated with the maturation of presynaptic proteins. However, we cannot exclude the possibility of reduced excitability or altered Ca^2+^ signaling in *maco-1* mutant neurons resulting in weak mislocalization of presynaptic proteins. Further studies on MACO-1 targets may reveal its molecular function and involvement in several intracellular events, such as the maturation of presynaptic proteins and proper neuronal activity.

## Materials and Methods

### Strains

*C. elegans* strains were maintained and grown according to standard procedures [42]. The following strains were used: wild-type Bristol strain (N2), wild-type Hawaiian strain (CB4856) for mapping with the snip-SNPs method [26], IK732 *maco-1(nj21)* I, IK734 *maco-1(nj34)* I, IK809 *maco-1(tm2917)* I, IK811 *maco-1(ok3165)* I, N2; *Is[flp-13p::snb-1::gfp]* II [41], and many transgenic strains derived from them. *maco-1(nj21)* and *maco-1(nj34)* mutants were isolated in genetic screens that bypassed temperature-dependent dauer formation of *daf-7(e1372)* backgrounds [43]. All mutant strains used in this study were backcrossed to N2 one to ten times before characterization.

### Behavioral analysis

Animals were grown at 20°C for all behavioral assays in this study, unless otherwise described. The procedure for the thermotaxis assay using radial temperature gradients was based on previously published research by Mori and Ohshima [6] with some modification. Evaluation of thermotaxis was consistent with methods of Mohri et al. [7]. Thermotaxis of individual animals on a radial temperature gradient was evaluated using the following four phenotypic categories: animals that moved to the cold region (i.e., center of plate) were classified as ‘17’; animals that moved to the 20°C region were classified as ‘20’; animals that moved to warm regions (i.e., plate periphery) were classified as ‘25’; animals that moved to cold and warm regions were classified as ‘17/25’. Chemotaxis to odorants was assayed according to Bargmann et al. [44], except that the assay plates contained a slightly different medium (2% agar, 1 mM magnesium sulfate (MgSO_4_), 1 mM calcium chloride (CaCl_2_) and 25 mM potassium phosphate (pH 6)). Dilutions of odorants with ethanol were as follows: 1∶1000 diacetyl, 1 mg/ml pyrazine, 1∶100 isoamyl alcohol, and 1∶100 benzaldehyde. The chemotaxis index was calculated according to Bargmann et al. [44]. Chemotaxis to NaCl was essentially as described previously [15]. Briefly, animals were scored as ‘normal’ when they migrated repeatedly to the concentration peak or remained at the peak during the assay. The animal was scored as ‘partially defective’ when it migrated toward the peak only once or stayed very briefly at the peak. The animal was scored as ‘defective’ when it failed to migrate to the peak.

### Molecular biology

The *maco-1 promoter::GFP* (pMYA3) used in Figure S3A was constructed by PCR amplification of the *maco-1* promoter region (i.e., the 4900-bp upstream sequence of *maco-1*). This fragment was then inserted into the GFP vector pPD95.77 to generate pMYA3. The *almost full-length genomic maco-1::GFP* (pMYA52) was constructed by PCR amplification of the 5′ 5-kilobase (kb) region of *maco-1* gene and 2-kb fragment downstream of 5′ 5-kb fragment using the cosmid C09C9 and wild-type genomic DNA, respectively. These fragments were inserted between the *maco-1* promoter and GFP of pMYA3 to generate the *almost full-length genomic maco-1::GFP* (pMYA52). The *almost full-length genomic maco-1* contains a *maco-1* promoter region and a 6621-bp fragment of a 7536-bp full-length genomic *maco-1* sequence, which includes predicted transmembrane domains and coiled coil domains. The full-length *maco-1* cDNA was constructed by PCR amplifications of the 5′ partial *maco-1* cDNA (723-bp region of 2694-bp full-length *maco-1* cDNA) and the rest of *maco-1* cDNA region using a cDNA library and yk1296a05 clone, respectively. These fragments were ligated into pPD49.26 to generate pMYA17. All *cell-specific promoter::maco-1* cDNA plasmids were generated by inserting each cell-specific promoter fragment into pMYA17. Cell-specific promoters are as follows: *unc-14p* for almost all neurons [45]; *gcy-8p* and *gcy-18p* for AFD neurons [17], [46]; *ttx-3p* for AIY neurons [43]; *lin-11p* for AIZ neurons (unpublished data); *odr-1p* for AWC neurons [47] and *glr-3p* for RIA neurons (unpublished data). Detailed expression patterns of these cell-specific promoters are shown in Table S2. The fluorescent protein fused *maco-1* cDNA and several organelle markers used in Figure 3 were also constructed. Specifically, the *glr-1* promoter of *glr-1p::YFP::TRAM* [29] was replaced with *gcy-8* promoter to generate *gcy-8p::YFP::TRAM* (pMYA161). The TRAM of pMYA161 was replaced with *maco-1* cDNA to generate *gcy-8p::YFP::maco-1* (pMYA174). The YFP of pMYA161 was replaced with CFP to generate *gcy-8p::CFP::TRAM* (pMYA212). The TRAM of pMYA161 was replaced with *FLJ10747* cDNA to generate *gcy-8p::YFP::FLJ10747* (pMYA182). The *glr-1* promoter of *glr-1p::MANS::YFP* [29] was replaced with *gcy-8* promoter to generate *gcy-8p::MANS::YFP* (pMYA145), and then YFP of pMYA145 was replaced with CFP to generate *gcy-8p::MANS::CFP* (pMYA208). The *glr-1* promoter of *glr-1p::YFP::GPI* [29] was replaced with the *gcy-8* promoter to generate *gcy-8p::YFP::GPI* (pMYA153). The *glr-1* promoter of *glr-1p::CFP::PIS* [29] was replaced with the *gcy-8* promoter to generate *gcy-8p::CFP::PIS* (pMYA141). The PIS of pMYA141 was replaced with *maco-1* cDNA to generate *gcy-8p::CFP::maco-1* (pMYA176). Two types of truncated forms of MACO-1, MACO-1 TM (1–453 amino acid) and MACO-1 CC (446–897 amino acid) (Figure 4), were PCR amplified from *unc-14p::maco-1 cDNA* and replaced with full-length *maco-1* cDNA of *unc-14p::maco-1* to generate *unc-14p::MACO-1 TM* (pMYA107) and *unc-14p::MACO-1 CC* (pMYA109), respectively. The *unc-14p::FLJ10747* shown in Figure 4 was constructed by ligation of the *unc-14* promoter into pPD49.26 vector to generate pMYA171. The full-length cDNA of human macoilin, FLJ10747 cDNA, was PCR amplified from a Mammalian Gene Collection (MGC) clone (Invitrogen), which contained full-length FLJ10747 cDNA. This product was inserted into pMYA171 to generate *unc-14p::FLJ10747* (pMYA172).

### Germline transformation

Transgenic strains were constructed by injecting DNA into the gonad of adult animals [48]. The *almost full-length genomic maco-1::GFP* used in Figure 3A, 3B and Figure S2A was injected into wild-type animals at a concentration of 50 ng/µl. Transgenic animals were recognized with neuronal GFP expression. Figure 3C–3E depicts the co-injection of *gcy-8p::YFP::maco-1* (pMYA174) (20 ng/µl) and *gcy-8p::CFP::TRAM* (pMYA212) (20 ng/µl) into wild-type animals. This injection was coupled with 120 ng/µl of pTAN124.5 (*ges-1p::DsRed*) as a co-injection marker. Figure 3F–3H shows that *gcy-8p::YFP::FLJ10747* (pMYA182) (50 ng/µl) and *gcy-8p::CFP::TRAM* (pMYA212) (4 ng/µl) were co-injected into wild-type animals with *ges-1p::DsRed* (pTAN124.5) (120 ng/µl) as a co-injection marker. Figure 4D shows co-injections of test DNA and the injection marker, *ges-1p::NLS-GFP* (pKDK66) [49], into the strain IK734, *maco-1(nj34)* at 2 and 50 ng/µl, respectively.

### Video analysis

Animal behavior was recorded using a custom made tracking and live imaging system. Well-fed single animal was placed on a TTX plate for tracking experiments. An ordinal analog video camera (Olympus) captured freely moving individual animal for 30 min with adequate sampling rate (30 frames/sec). A computer-controlled microscope stage was automatically moved to center animals in the visual field using a custom image analysis algorithm with microscope software (MetaMorph: Universal Imaging Corporation). Midlines of recorded animals were extracted from each image, and curvatures at certain positions were calculated by analyzing points along midline with constant intervals. Spatio-temporal curvature maps were constructed by expressing the quantity of curvatures with a color-map (x-axis = time and y-axis = position along midline of animal). A smoothing filter (3×3 dimensions) was applied to the curvature map to reduce noise effects. Omega turn states (i.e., head of the animal curls to touch its own body; Figure 5B) were identified by an independent algorithm and expressed as white color in the curvature map. All video images were analyzed with MATLAB (Mathworks, Natick, MA).

### *In vivo* calcium imaging and data analysis

*In vivo* calcium imaging was performed according to previous studies [21], [50]. Well-fed animals expressing yellow cameleon 3.60 [51] caused by AFD and AIY promoters, *gcy-8p::yc3.60* (pSAS309) and *AIYp::yc3.60* (pNR86.79), were glued onto a 2% agar pad on glass, immersed in M9 buffer, and covered by cover glass. The agar pad and M9 buffer were kept at the initial imaging temperature. Sample preparation was completed within one minute. The sample was then placed onto a Peltier-based thermocontroller (Tokai Hit, Japan) on the stage of a Nikon E600 or Olympus BX61WI microscope at the initial imaging temperature for two minutes. Fluorescence was introduced into W-View (Hamamatsu Photonics, Japan) or Dual-View (Molecular devices, USA) optic systems. Cyan fluorescent protein (CFP; F480) and yellow fluorescent protein (YFP; F535) images were simultaneously captured by a CCD camera HiSCA or EM-CCD camera C9000-13 ImagEM (Hamamatsu Photonics). Images were taken with a 200-ms exposure time with 4×4 or 2×2 binning. Temperatures on the agar pad were monitored by the thermometer system, DCM-20 (Tokai Hit and Hamamatsu Photonics). For each imaging experiment, fluorescence intensities of F535 and F480 were measured using AquaCosmos (Hamamatsu Photonics) or MetaMorph (Molecular Device) imaging analysis systems. Relative increases or decreases in intracellular Ca^2+^ concentrations were measured as increases or decreases in YFP/CFP fluorescence ratios of cameleon protein (ratio change).

### Statistical analysis

Error bars in figures indicate standard errors (SEM). The statistical analysis for behavioral experiments was performed by one-way analysis of variance (ANOVA) for multiple comparisons followed by post hoc Dunnett's multiple comparison. A single asterisk and double asterisk indicate statistical significance at p<0.05 and p<0.01, respectively. The value of p is probability.

## Supporting Information

Figure S1 Chemotaxis to odorants. (A) Chemotaxis to diacetyl with varying dilutions. (B) Chemotaxis to pyrazine with varying dilutions. (C) Chemotaxis to isoamyl alcohol with varying dilutions. (D) Chemotaxis to benzaldehyde with varying dilutions. Error bar indicates the standard error of the mean (SEM). Asterisks indicate statistically significant differences between the index of *maco-1(nj21)* or *maco-1(nj34)* and index of wild-type at each concentration. One asterisk denotes statistical significance at the p<0.05 level and two asterisks denote statistical significance at the p<0.01 level (ANOVA with a Dunnett's post hoc test).
(0.26 MB TIF)

Figure S2 Rescue experiments for thermotaxis defects in *maco-1(nj34)* mutants. (A–C) All animals tested were cultivated at 20°C. The y-axis shows the fraction of 20 animals that migrated to the 20°C region. Error bar indicates the standard error of the mean (SEM). (A) Expression of *almost full-length genomic maco-1::GFP* (50 ng/µl) in most neurons of *maco-1(nj34)* mutants rescued the abnormal thermotaxis phenotype of *maco-1*. Statistical significance could not be described due to insufficient assay numbers (two). However, there was an obvious difference between the fraction of 20 of *maco-1(nj34)* and that of *maco-1(nj34)*; *Ex[almost full-length genomic maco-1::GFP]* strain (n = 40 animals; 20 animals per trial). (B) Rescue experiments with varying concentration of FLJ10747, human *maco-1* cDNA (n = 99–260 animals). Single and double asterisks indicate fractions of 20 of each transgenic strain that was different from fraction of 20 of *maco-1(nj34)* mutants at the p<0.05 and p<0.01 level, respectively (ANOVA with a Dunnett's post hoc test). (C) n = 60–120 animals. Double asterisks indicate the fraction of 20 of *maco-1(nj34)*; *Ex[unc-14p::YFP::maco-1]* strain that were different from that of *maco-1(nj34)* mutants at the p<0.01 level (ANOVA with a Dunnett's post hoc test).
(0.34 MB TIF)

Figure S3 Expression pattern of MACO-1 and neuronal morphology of AFD thermosensory neurons and AIY ineterneurons. (A) Expression of *maco-1 promoter::GFP* (pMYA3) in wild-type. DIC and GFP images are merged. Anterior is to the left. GFP expression was observed in many neurons. Names of several neurons are shown. (B, C) Expression of GFP in AFD and AIY neurons. (B) Wild-type. (C) *maco-1* mutants. Left panels show merged DIC and GFP images and right panels are GFP images alone. A yellow arrowhead and white arrowhead indicate the cell body of AFD and AIY neurons, respectively. Yellow arrows show dendrites (left side of the yellow arrowhead) and axons (right side of the yellow arrowhead) of AFD neurons. A white arrow shows an axon of AIY neurons. Scale bars = 5 µm.
(1.93 MB TIF)

Figure S4 Subcellular localization of yellow fluorescent protein (YFP)::MACO-1, YFP::MACO-1 TM, and YFP::MACO-1 CC. (A–C) Expression of YFP::MACO-1 in almost all neurons and cyan fluorescent protein (CFP)::TRAM (rER marker) in AFD neurons of *maco-1(nj34)* animals. This transgenic strain showed a partial-rescued phenotype (Figure S2C). (A) YFP::MACO-1. (B) CFP::TRAM. (C) Merged YFP::MACO-1 and CFP::TRAM images. YFP::MACO-1 was localized to peri-nuclear regions and co-localized with CFP::TRAM, suggesting that MACO-1 is localized to the rER. (D–F) Expression of YFP::MACO-1 TM and CFP::TRAM in AFD neuron of wild-type animals. (D) YFP::MACO-1 TM. (E) CFP::TRAM. (F) Merged YFP::MACO-1 TM and CFP::TRAM images. YFP::MACO-1 TM was co-localized with CFP::TRAM, suggesting that MACO-1 TM is localized to the rER. (G–I) Expression of YFP::MACO-1 CC and CFP::TRAM in AFD neuron among wild-type animals. (G) YFP::MACO-1 CC. (H) CFP::TRAM. (I) Merged YFP::MACO-1 CC and CFP::TRAM images. YFP::MACO-1 CC was negligibly co-localized with CFP::TRAM, suggesting that MACO-1 CC is only slightly localized to the rER. (J, K) Expression of YFP::MACO-1 TM in almost all neurons in *maco-1(nj34)*. This strain did not show the rescued phenotype (Figure S2C). (J) Merged DIC and YFP images. (K) YFP image. (L, M) Expression of YFP::MACO-1 CC in almost all neurons in *maco-1(nj34)*. This strain showed little to no rescued phenotype (Figure S2C). (L) Merged DIC and YFP images. (M) YFP image. Dashed ellipse shows a nerve ring (i.e., ring-shaped zone where axons of many neurons overlap). YFP::MACO-1 CC is localized to not only cytoplasm but neurites. Adult animals were observed in all images. Scale bars: A–C and J–M = 5 µm; D–I = 2 µm.
(3.51 MB TIF)

Figure S5 Slight mislocalization of presynaptic marker, SNB-1::GFP, in *maco-1* mutants. (A–D) SNB-1::GFP expression in six DD motor neurons of L2 larva. We observed transgenic strains, N2; Is[*flp-13p::SNB-1::GFP*] [41] and *maco-1(nj34)*; Is[*flp-13p::SNB-1::GFP*]. The expression pattern of *flp-13p::GFP* was shown in Table S2 [40]. Scale bars = 5 µm. (A) Wild-type. (B) *maco-1(nj34)* mutant. (C, D) Arrowheads show cell bodies of DD2, DD3 and DD4 motor neurons. (E) Quantification of distance between adjacent puncta. We measured the distance between adjacent puncta and categorized it into sixteen classes. The x-axis and y-axis show each class and fraction of distance categorized into one of sixteen classes, respectively. Error bar indicates the standard error of the mean (SEM). The statistical difference was determined by using a two-tailed Student's test. A single asterisk indicates statistically significant difference at p<0.05. n = 27 (wild-type) and 30 (*maco-1*).
(1.42 MB TIF)

Table S1 Homology search. Results of a homology search by Blast using amino acid sequences of MACO-1 or human homolog of MACO-1 as queries. Several molecules of vertebrate or invertebrate are shown for each survey. The identity and similarity are calculated using the software program, Mac vector.
(0.04 MB DOC)

Table S2 Expression patterns driven by each promoter. We used *lin-11p* as an AIZ promoter, *glr-3p* as a RIA promoter and *odr-1p* as an AWC promoter (Figure 4D). Detailed information about expression patterns of *unc-14p*, *gcy-8p*, *gcy-18p*, *ttx-3p*, *odr-1p* and *flp-13p* are shown in previous studies [17], [40], [43], [45]–[47]. Data describing expression patterns of *lin-11p* or *glr-3p* are unpublished.
(0.03 MB DOC)

Text S1 Supplemental Materials and Methods.
(0.05 MB DOC)
