# Supplementary figures and images for: Novel and Conserved Protein Macoilin Is Required for Diverse Neuronal Functions in Caenorhabditis elegans
Source: PLoS Genet. 2011 May 12;7(5):e1001384. doi: 10.1371/journal.pgen.1001384 (PMC3093358; doi:10.1371/journal.pgen.1001384)

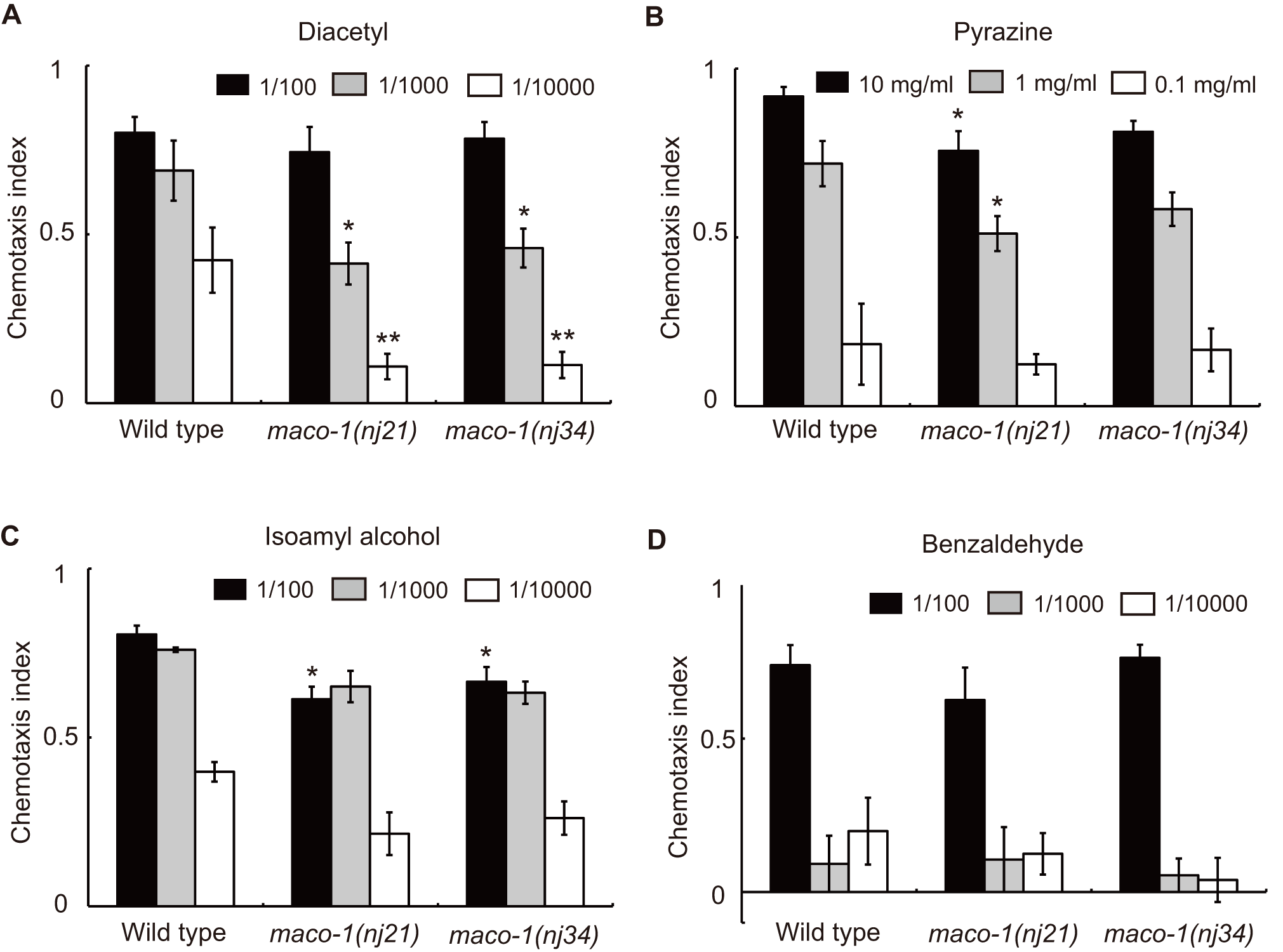

Supplement: Figure S1 — Chemotaxis to odorants. (A) Chemotaxis to diacetyl with varying dilutions. (B) Chemotaxis to pyrazine with varying dilutions. (C) Chemotaxis to isoamyl alcohol with varying dilutions. (D) Chemotaxis to benzaldehyde with varying dilutions. Error bar indicates the standard error of the mean (SEM). Asterisks indicate statistically significant differences between the index of maco-1(nj21) or maco-1(nj34) and index of wild-type at each concentration. One asterisk denotes statistical significance at the p<0.05 level and two asterisks denote statistical significance at the p<0.01 level (ANOVA with a Dunnett's post hoc test). (0.26 MB TIF) [file pgen.1001384.s001.tif]

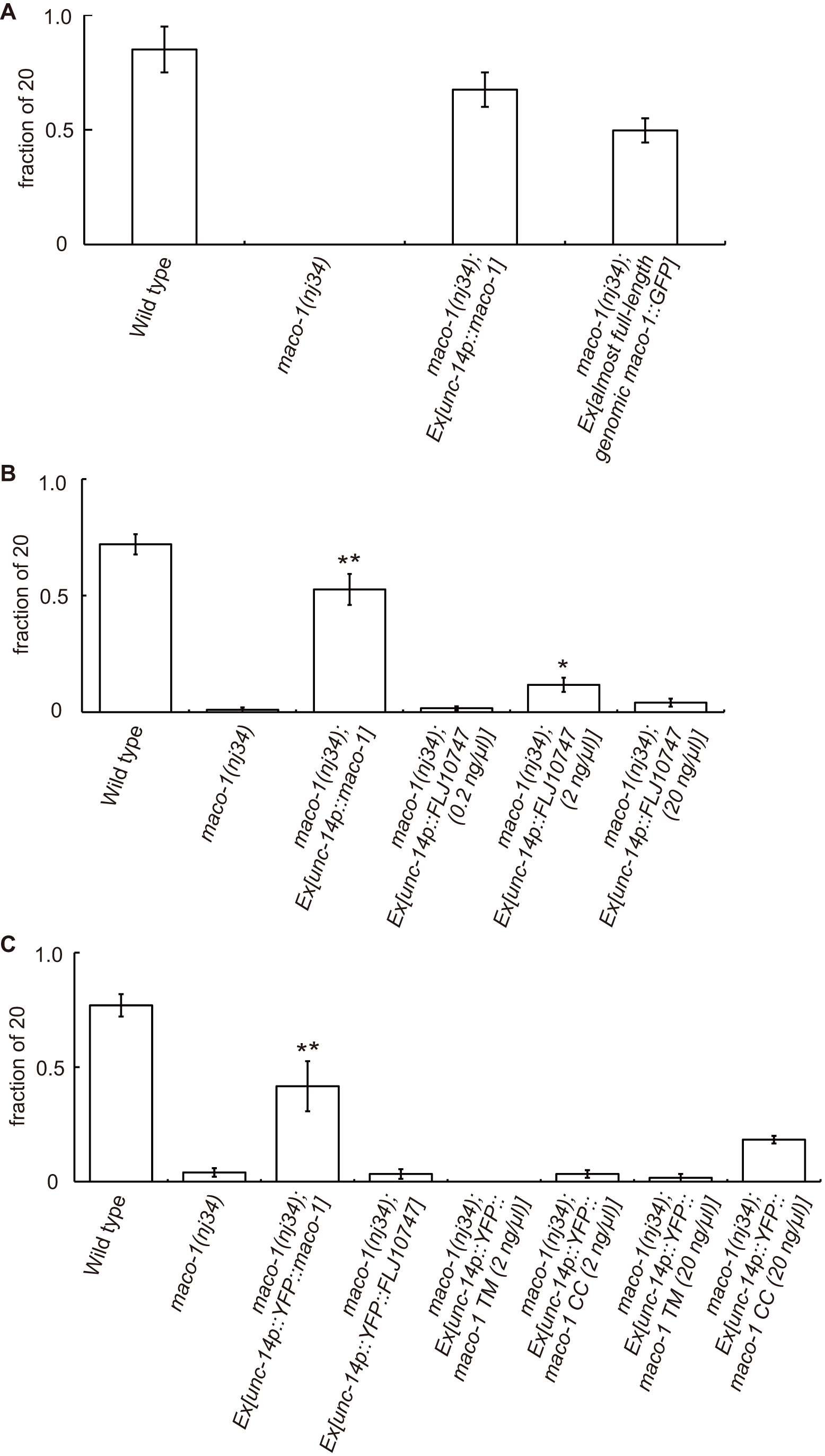

Supplement: Figure S2 — Rescue experiments for thermotaxis defects in maco-1(nj34) mutants. (A–C) All animals tested were cultivated at 20°C. The y-axis shows the fraction of 20 animals that migrated to the 20°C region. Error bar indicates the standard error of the mean (SEM). (A) Expression of almost full-length genomic maco-1::GFP (50 ng/µl) in most neurons of maco-1(nj34) mutants rescued the abnormal thermotaxis phenotype of maco-1. Statistical significance could not be described due to insufficient assay numbers (two). However, there was an obvious difference between the fraction of 20 of maco-1(nj34) and that of maco-1(nj34); Ex[almost full-length genomic maco-1::GFP] strain (n = 40 animals; 20 animals per trial). (B) Rescue experiments with varying concentration of FLJ10747, human maco-1 cDNA (n = 99–260 animals). Single and double asterisks indicate fractions of 20 of each transgenic strain that was different from fraction of 20 of maco-1(nj34) mutants at the p<0.05 and p<0.01 level, respectively (ANOVA with a Dunnett's post hoc test). (C) n = 60–120 animals. Double asterisks indicate the fraction of 20 of maco-1(nj34); Ex[unc-14p::YFP::maco-1] strain that were different from that of maco-1(nj34) mutants at the p<0.01 level (ANOVA with a Dunnett's post hoc test). (0.34 MB TIF) [file pgen.1001384.s002.tif]

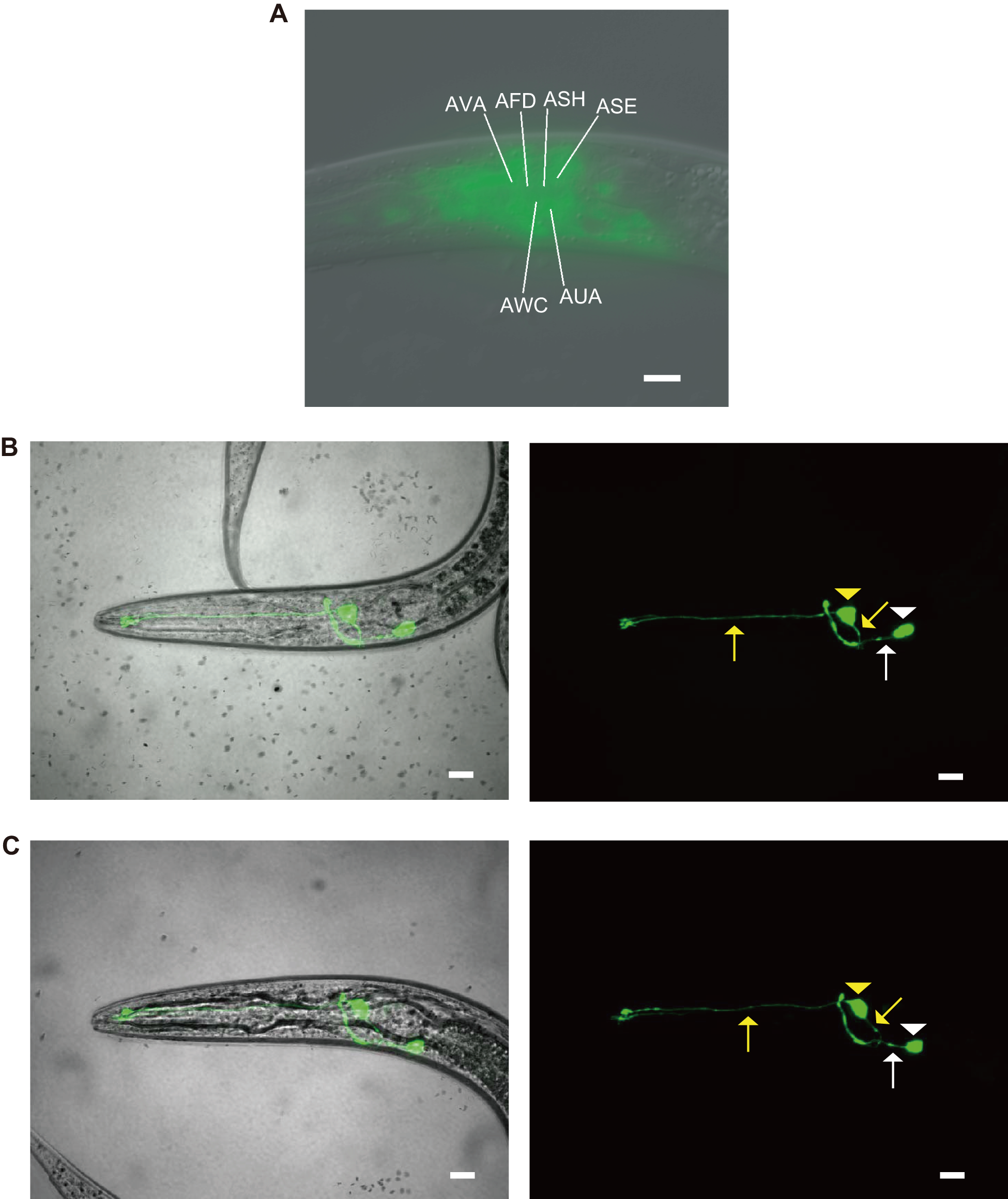

Supplement: Figure S3 — Expression pattern of MACO-1 and neuronal morphology of AFD thermosensory neurons and AIY ineterneurons. (A) Expression of maco-1 promoter::GFP (pMYA3) in wild-type. DIC and GFP images are merged. Anterior is to the left. GFP expression was observed in many neurons. Names of several neurons are shown. (B, C) Expression of GFP in AFD and AIY neurons. (B) Wild-type. (C) maco-1 mutants. Left panels show merged DIC and GFP images and right panels are GFP images alone. A yellow arrowhead and white arrowhead indicate the cell body of AFD and AIY neurons, respectively. Yellow arrows show dendrites (left side of the yellow arrowhead) and axons (right side of the yellow arrowhead) of AFD neurons. A white arrow shows an axon of AIY neurons. Scale bars = 5 µm. (1.93 MB TIF) [file pgen.1001384.s003.tif]

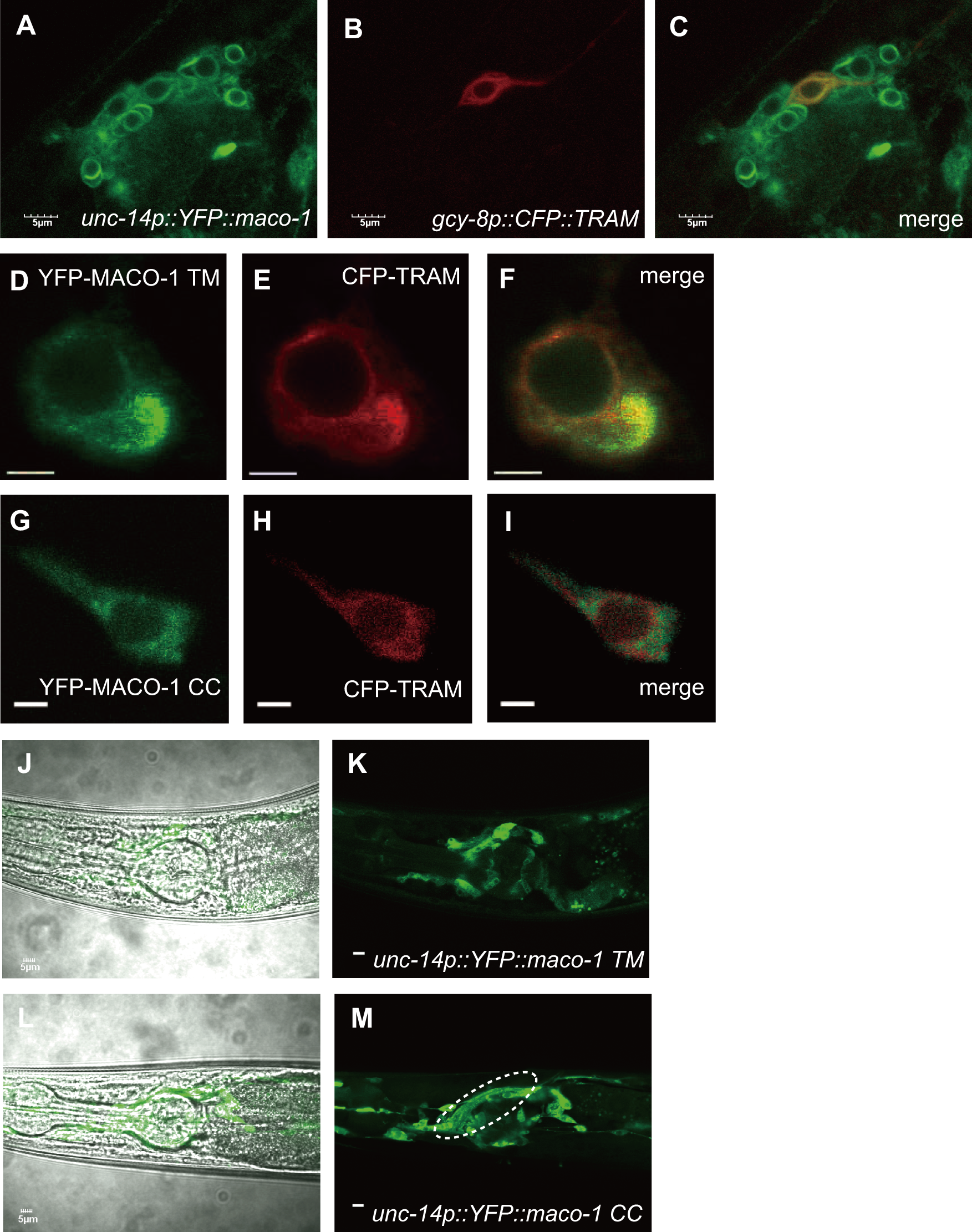

Supplement: Figure S4 — Subcellular localization of yellow fluorescent protein (YFP)::MACO-1, YFP::MACO-1 TM, and YFP::MACO-1 CC. (A–C) Expression of YFP::MACO-1 in almost all neurons and cyan fluorescent protein (CFP)::TRAM (rER marker) in AFD neurons of maco-1(nj34) animals. This transgenic strain showed a partial-rescued phenotype (Figure S2C). (A) YFP::MACO-1. (B) CFP::TRAM. (C) Merged YFP::MACO-1 and CFP::TRAM images. YFP::MACO-1 was localized to peri-nuclear regions and co-localized with CFP::TRAM, suggesting that MACO-1 is localized to the rER. (D–F) Expression of YFP::MACO-1 TM and CFP::TRAM in AFD neuron of wild-type animals. (D) YFP::MACO-1 TM. (E) CFP::TRAM. (F) Merged YFP::MACO-1 TM and CFP::TRAM images. YFP::MACO-1 TM was co-localized with CFP::TRAM, suggesting that MACO-1 TM is localized to the rER. (G–I) Expression of YFP::MACO-1 CC and CFP::TRAM in AFD neuron among wild-type animals. (G) YFP::MACO-1 CC. (H) CFP::TRAM. (I) Merged YFP::MACO-1 CC and CFP::TRAM images. YFP::MACO-1 CC was negligibly co-localized with CFP::TRAM, suggesting that MACO-1 CC is only slightly localized to the rER. (J, K) Expression of YFP::MACO-1 TM in almost all neurons in maco-1(nj34). This strain did not show the rescued phenotype (Figure S2C). (J) Merged DIC and YFP images. (K) YFP image. (L, M) Expression of YFP::MACO-1 CC in almost all neurons in maco-1(nj34). This strain showed little to no rescued phenotype (Figure S2C). (L) Merged DIC and YFP images. (M) YFP image. Dashed ellipse shows a nerve ring (i.e., ring-shaped zone where axons of many neurons overlap). YFP::MACO-1 CC is localized to not only cytoplasm but neurites. Adult animals were observed in all images. Scale bars: A–C and J–M = 5 µm; D–I = 2 µm. (3.51 MB TIF) [file pgen.1001384.s004.tif]

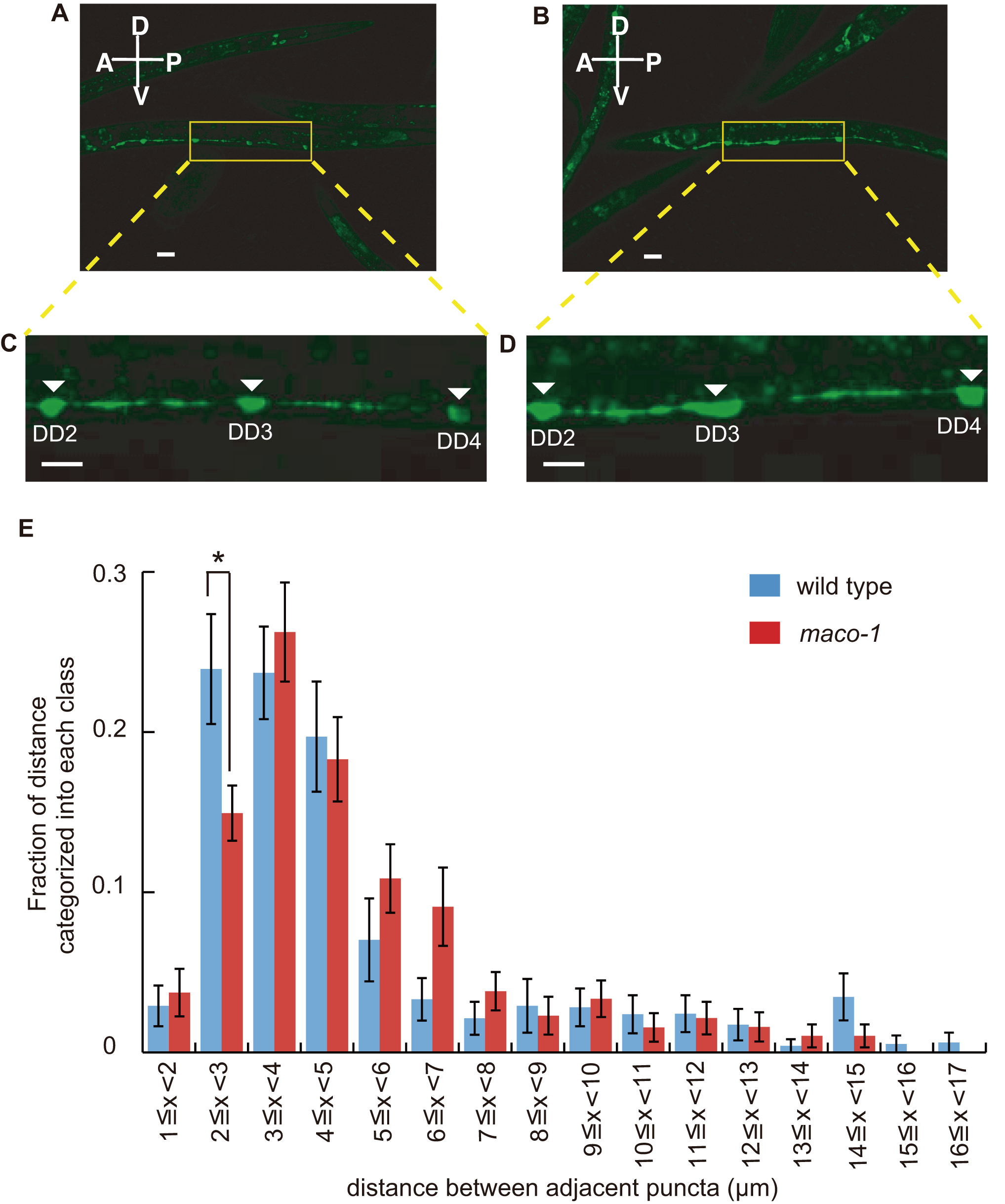

Supplement: Figure S5 — Slight mislocalization of presynaptic marker, SNB-1::GFP, in maco-1 mutants. (A–D) SNB-1::GFP expression in six DD motor neurons of L2 larva. We observed transgenic strains, N2; Is[flp-13p::SNB-1::GFP] [41] and maco-1(nj34); Is[flp-13p::SNB-1::GFP]. The expression pattern of flp-13p::GFP was shown in Table S2 [40]. Scale bars = 5 µm. (A) Wild-type. (B) maco-1(nj34) mutant. (C, D) Arrowheads show cell bodies of DD2, DD3 and DD4 motor neurons. (E) Quantification of distance between adjacent puncta. We measured the distance between adjacent puncta and categorized it into sixteen classes. The x-axis and y-axis show each class and fraction of distance categorized into one of sixteen classes, respectively. Error bar indicates the standard error of the mean (SEM). The statistical difference was determined by using a two-tailed Student's test. A single asterisk indicates statistically significant difference at p<0.05. n = 27 (wild-type) and 30 (maco-1). (1.42 MB TIF) [file pgen.1001384.s005.tif]
