# Supplementary material for: Novel and Conserved Protein Macoilin Is Required for Diverse Neuronal Functions in Caenorhabditis elegans
Source: PLoS Genet. 2011 May 12;7(5):e1001384. doi: 10.1371/journal.pgen.1001384 (PMC3093358; doi:10.1371/journal.pgen.1001384)
Supplement: Table S1 — Homology search. Results of a homology search by Blast using amino acid sequences of MACO-1 or human homolog of MACO-1 as queries. Several molecules of vertebrate or invertebrate are shown for each survey. The identity and similarity are calculated using the software program, Mac vector. (0.04 MB DOC) [file pgen.1001384.s006.doc]

| Sequence | Species | E-value | Identity | | | Similarity |
| --- | --- | --- | --- | --- | --- | --- |
| Query = MACO-1 (*Caenorhabditis elegans*) | | | | | | |
| transmembrane proteins 57 | *Mus musculus* | 2e-40 | | 18% | 32% | |
| Transmembrane protein 57 | *Homo sapiens* | 5e-39 | | 18% | 32% | |
| CG30389 | *Drosophila melanogaster* | 3e-26 | | 18% | 34% | |
| macoilin | *Xenopus laevis* | 1e-24 | 17% | | 31% | |
| Query = transmembrane protein 57 (*Homo sapiens*) | | | | | | |
| transmembrane proteins 57 | *Mus musculus* | 0 | 99% | | 99% | |
| macoilin | *Xenopus laevis* | 0 | 89% | | 95% | |
| CG30389 | *Drosophila melanogaster* | 9e-102 | 29% | | 41% | |
| hypothetical protein D2092.5 (MACO-1) | *Caenorhabditis elegans* | 2e-24 | 18% | | 32% | |
