# Supplementary material for: Novel and Conserved Protein Macoilin Is Required for Diverse Neuronal Functions in Caenorhabditis elegans
Source: PLoS Genet. 2011 May 12;7(5):e1001384. doi: 10.1371/journal.pgen.1001384 (PMC3093358; doi:10.1371/journal.pgen.1001384)
Supplement: Table S2 — Expression patterns driven by each promoter. We used lin-11p as an AIZ promoter, glr-3p as a RIA promoter and odr-1p as an AWC promoter (Figure 4D). Detailed information about expression patterns of unc-14p, gcy-8p, gcy-18p, ttx-3p, odr-1p and flp-13p are shown in previous studies [17], [40], [43], [45]–[47]. Data describing expression patterns of lin-11p or glr-3p are unpublished. (0.03 MB DOC) [file pgen.1001384.s007.doc]

| promoter | Expression pattern (*promoter::gfp*) |
| --- | --- |
| *unc-14p* | almost all neurons |
| AFDp | AFD |
| AIYp | AIY |
| *lin-11p* | ADF, ADL, **AIZ**, AVG, AVH, AVJ, RIC |
| *glr-3p* | **RIA**, ASE |
| *odr-1p* | AWB, **AWC** |
| *flp-13p* | ASE, ASG, ASK, BAG, DD, I5, M3, M5 |
