# Supplementary material for: Novel and Conserved Protein Macoilin Is Required for Diverse Neuronal Functions in Caenorhabditis elegans
Source: PLoS Genet. 2011 May 12;7(5):e1001384. doi: 10.1371/journal.pgen.1001384 (PMC3093358; doi:10.1371/journal.pgen.1001384)
Supplement: Text S1 — Supplemental Materials and Methods. (0.05 MB DOC) [file pgen.1001384.s008.doc]

**Supplemental Materials and Methods**

**Molecular biology**

The *unc-14p::YFP::maco-1* (pMYA204) used in Figure S2 and S4 was constructed by replacing the *glr-1* promoter of *glr-1p::YFP::TRAM* [29] with the *ttx-3* promoter to generate *ttx-3p::YFP::TRAM* (pMYA163). The *maco-1* cDNA was amplified by PCR and replaced with the TRAM of pMYA163 to generate *ttx-3p::YFP::maco-1* (pMYA178). The *ttx-3* promoter of pMYA178 was replaced with *unc-14* promoter to generate *unc-14p::YFP::maco-1* (pMYA204). The *unc-14p::YFP::FLJ10747* (pMYA200) used in Figure S2 was constructed by PCR amplification of the FLJ10747 cDNA. This construct was replaced with the TRAM of *ttx-3p::YFP::TRAM* (pMYA163) to generate *ttx-3p::YFP::FLJ10747* (pMYA186). The *ttx-3* promoter of pMYA186 was replaced with *unc-14* promoter to generate *unc-14p::YFP::FLJ10747* (pMYA200). The *unc-14p::YFP::MACO-1 TM* (pMYA216) used in Figure S2 and S4 was constructed by PCR amplification of the MACO-1 TM region (from 1 to 453 amino acids) from *maco-1* cDNA. This construct was replacing with the TRAM of *ttx-3p::YFP::TRAM* (pMYA163) to generate *ttx-3p::YFP::MACO-1 TM* (pMYA121). The *ttx-3* promoter of pMYA121 was replaced with *unc-14* promoter to generate *unc-14p::YFP::MACO-1 TM* (pMYA216). The *unc-14p::YFP::MACO-1 CC* (pMYA218) used in Figure S2 and S4 was constructed by PCR amplification of the MACO-1 CC region (from 446 to 897 amino acids) from *maco-1* cDNA. This construct was replacing with the TRAM of *ttx-3p::YFP::TRAM* (pMYA163) to generate *ttx-3p::YFP::MACO-1 CC* (pMYA238). The *ttx-3* promoter of pMYA238 was replaced with *unc-14* promoter to generate *unc-14p::YFP::MACO-1 CC* (pMYA218). The *gcy-8p::YFP::MACO-1 TM* (pMYA119) used in Figure S4 was constructed by PCR amplification of the MACO-1 TM region (from 1 to 453 amino acids) from *maco-1* cDNA. This construct was replacing with the TRAM of *gcy-8p::YFP::TRAM* (pMYA161) to generate *gcy-8p::YFP::MACO-1 TM* (pMYA119). The *gcy-8p::YFP::MACO-1 CC* (pMYA236) used in Figure S4 was constructed by PCR amplification of the MACO-1 CC region (from 446 to 897 amino acids) from *maco-1* cDNA. This construct was replacing with the TRAM of *gcy-8p::YFP::TRAM* (pMYA161) to generate *gcy-8p::YFP::MACO-1 CC* (pMYA236).

**Germline transformation**

Transgenic strains were constructed by injecting DNA into the gonads of adult animals [48]. The *unc-14p::FLJ10747* (pMYA172) was injected at a concentration of 0.2, 2 or 20 ng/μl and an injection marker *ges-1p::NLS-GFP* (pKDK66) was co-injected into the strain IK734, *maco-1(nj34)* at a concentration of 50 ng/μl (Figure S2B). The *unc-14p::YFP::maco-1* (pMYA204) (20 ng/μl) and *gcy-8p::CFP::TRAM* (pMYA212) (4 ng/μl) were co-injected into *maco-1* with 120 ng/μl of *ges-1p::DsRed* (pTAN124.5) as a co-injection marker (Figure S2C). The *unc-14p::YFP::FLJ10747* (pMYA200) (20 ng/μl) and *gcy-8p::CFP::TRAM* (pMYA212) (4 ng/μl) were co-injected into *maco-1* with 120 ng/μl of *ges-1p::DsRed* (pTAN124.5) as a co-injection marker (Figure S2C). The *unc-14p::YFP::MACO-1 TM* (pMYA216) (2 or 20 ng/μl) or *unc-14p::YFP::MACO-1 CC* (pMYA218) (2 or 20 ng/μl) and *gcy-8p::CFP::TRAM* (pMYA212) (4 ng/μl) were co-injected into *maco-1* with 120 ng/μl of *ges-1p::DsRed* (pTAN124.5) as a co-injection marker (Figure S2C). The *maco-1p::GFP* (pMYA3) was injected into wild-type animals at a concentration of 50 ng/μl (Figure S3A)*.* The transgenic arrays of N2; Ex[*H13p (AFDp)::GFP* (30 ng/μl), *AIYp::GFP* (20 ng/μl)] (i.e., IK49) strain were transferred in *maco-1* mutant animals by intercrossing to make the following transgenic strain, *maco-1(nj34)*; Ex[*H13p (AFDp)::GFP* (30 ng/μl), *AIYp::GFP* (20 ng/μl)] (Figure S3C). The *unc-14p::YFP::MACO-1* (pMYA204) (20 ng/μl) and *gcy-8p::CFP::TRAM* (pMYA212) (4 ng/μl) were co-injected into *maco-1* with 120 ng/μl of *ges-1p::DsRed* (pTAN124.5) as a co-injection marker (Figure S4A-C). The *gcy-8p::YFP::MACO-1 TM* (pMYA119) (20 ng/μl) and *gcy-8p::CFP::TRAM* (pMYA212) (4 ng/μl) were co-injected into wild-type animals with 120 ng/μl of *ges-1p::DsRed* (pTAN124.5) as a co-injection marker (Figure S4D-F). The *gcy-8p::YFP::MACO-1 CC* (pMYA236) (20 ng/μl) and *gcy-8p::CFP::TRAM* (pMYA212) (4 ng/μl) were co-injected into wild-type animals with 120 ng/μl of *ges-1p::DsRed* (pTAN124.5) as a co-injection marker (Figure S4G-I). The *unc-14p::YFP::MACO-1 TM* (pMYA216) (20 ng/μl) and *gcy-8p::CFP::TRAM* (pMYA212) (4 ng/μl) were co-injected into wild-type animals with 120 ng/μl of *ges-1p::DsRed* (pTAN124.5) as a co-injection marker (Figure S4J and S4K). The *unc-14p::YFP::MACO-1 CC* (pMYA218) (20 ng/μl) and *gcy-8p::CFP::TRAM* (pMYA212) (4 ng/μl) were co-injected into wild-type animals with 120 ng/μl of *ges-1p::DsRed* (pTAN124.5) as a co-injection marker (Figure S4L and S4M).

**Green fluorescent protein (GFP) analysis**

For the GFP intensity quantification, images were taken with Fluoview FV1000 confocal laser scanning microscope (Olympus) (100X objective) under identical exposure conditions. Confocal stacks were projected into a single plane using Olympus Fluoview FV1000 software and exported as a tiff file. Files were converted to a binary image using the threshold command to resemble a red, green, blue (RGB) image. A region of interest was drawn around the relevant area of the nerve cord. The flp-13p::SNB-1::GFP localization in wild-type or *maco-1(nj34)* mutant animals was scored in ventral nerve cord of L2 animals. Distances between adjacent puncta within the ventral nerve cord were measured by MetaMorph (Universal Imaging Corporation). Each puncta was recognized using the ‘count nuclei’ tool, and the centroid of each puncta was determined by the ‘Integrated Morphometry Analysis’ tool in MetaMorph. Adjacent centroids of puncta were connected with a straight line by the ‘single line’ tool. Line lengths were measured as the distance between adjacent synapses using the ‘Region measurement’ tool in MetaMorph.

**Statistical analysis**

All error bars indicate the standard error of the mean (SEM). The statistical analysis for behavioral experiments was performed by one-way analysis of variance (ANOVA) for multiple comparisons followed by post hoc Dunnett’s multiple comparison (Figure S1 and S2). The statistical analysis for distance between puncta was performed by a two-tailed Student’s test (Figure S5E). A single asterisk and double asterisks indicate statistical significance at the p < 0.05 and p < 0.01 level, respectively. The value of p is probability.
